# Microglia in depression: an overview of microglia in the pathogenesis and treatment of depression

**DOI:** 10.1186/s12974-022-02492-0

**Published:** 2022-06-06

**Authors:** Haixia Wang, Yi He, Zuoli Sun, Siyu Ren, Mingxia Liu, Gang Wang, Jian Yang

**Affiliations:** 1 The National Clinical Research Center for Mental Disorders and Beijing Key Laboratory of Mental Disorders, Beijing Anding Hospital, Capital Medical University, 5 Ankang Lane, Dewai Avenue, Xicheng District, Beijing, 100088 China; 2 Advanced Innovation Center for Human Brain Protection, Capital Medical University, 10 Xi tou tiao, You An Men Wai, Fengtai District, Beijing, 100069 China

**Keywords:** Major depressive disorder, Microglial activation, Antidepressants, NLRP3 inflammasome, Neurogenesis, Kynurenine pathway

## Abstract

Major depressive disorder is a highly debilitating psychiatric disorder involving the dysfunction of different cell types in the brain. Microglia are the predominant resident immune cells in the brain and exhibit a critical role in depression. Recent studies have suggested that depression can be regarded as a microglial disease. Microglia regulate inflammation, synaptic plasticity, and the formation of neural networks, all of which affect depression. In this review, we highlighted the role of microglia in the pathology of depression. First, we described microglial activation in animal models and clinically depressed patients. Second, we emphasized the possible mechanisms by which microglia recognize depression-associated stress and regulate conditions. Third, we described how antidepressants (clinical medicines and natural products) affect microglial activation. Thus, this review aimed to objectively analyze the role of microglia in depression and focus on potential antidepressants. These data suggested that regulation of microglial actions might be a novel therapeutic strategy to counteract the adverse effects of devastating mental disorders.

**Supplementary Information:**

The online version contains supplementary material available at 10.1186/s12974-022-02492-0.

## Background

Major depressive disorder (MDD) is a public health problem that affects approximately 322 million people worldwide [1]. During the coronavirus disease 2019 (COVID-19) pandemic, a meta‐analysis reported that 45% of COVID‐19 patients experienced depression [2]. Over 700,000 people die from suicide every year due to depressive disorder, which results in a heavy burden on individuals and society [3]. Clinical symptoms of MDD include persistent low mood, appetite loss, decreased interest in favorite activities, despair, sleepy disorders, and, in severe cases, suicidal behavior [4]. Unfortunately, due to its complexity and heterogeneity that are determined by genetic and environmental factors, the biological mechanisms underlying depression remain unclear. Currently, theories concerning depression primarily focus on the monoamine neurotransmitter depletion hypothesis, neuroplasticity hypothesis, and hypothalamus–pituitary–adrenal (HPA) axis hypothesis [5]. However, limitations are associated with these specific pathological mechanisms, such as an inability to explain the delayed effect of antidepressants and a lack of focus on cells other than neurons in the central nervous system (CNS) [6, 7].

Microglia are the first line in the innate immune system in the CNS and actively regulate microenvironmental changes in healthy and disordered brains. Microglial activation regulates inflammation, synaptic refinement, synaptic pruning, and neuronal connectivity [8]. Depression is considered a microglia-associated disorder (microgliopathy) [9]. Ample evidence suggests that microglia-mediated neuroinflammation interacts with all three theories correlated with MDD listed above [10, 11]. Neuroinflammation and the HPA axis are thought to function in a coordinated manner, and their dysregulation might mediate the onset of depression [10, 12–14]. Some clinical antidepressants affect the activation of microglia and neuroinflammation [15–17]. Nonsteroidal anti-inflammatory drugs appear to alleviate depressive symptoms by inhibiting microglial activation [18]. Minocycline is a semisynthetic tetracycline antibiotic that decidedly improves depressive-like behaviors by inhibiting microglial activation in the prefrontal cortex (PFC) and hippocampus (HIP) [19]. Microglial activation can be divided into the classic activation of M1 or alternate activation of M2 under optimal conditions [20]. Recently, pharmacological principles that modulate microglial polarization may provide beneficial treatments to alleviate the recurrence of psychiatric disorders [21–23].

Therefore, we conducted a systematic review using the *Pubmed* electronic database. Search themes included *depression and microglia*, *neurogenesis*, *stress*, and *antidepressants*. Articles from 1992 to 2021 were reviewed that focused on the connection between microglia and depression. In this review, we described the activation states of microglia in animal models and clinically depressed patients. We elucidated the mechanisms underlying depression and the therapeutic potential of targeting microglia. Finally, we highlighted the protective effects of antidepressants that act through modulating microglia in stress-induced animal models of depression.

### Microglia: origin and function

#### Origin and development of microglia

Microglia were first described as a distinct cell type by Spanish neuroscientist *Pio del Rio-Hortega* in 1919 and account for approximately 5–10% of the total cell population in the brain [24]. The number of microglia in the adult mouse brain is estimated at 3.5 million, with variations in density across different regions. For example, more microglia are found in the HIP, substantia nigra (SN), olfactory telencephalon, and basal ganglia. Fewer microglia are observed in the fiber tracts, cerebellum, and the majority of brainstem [25]. Ontogenetically, recent fate-mapping studies have revealed that microglia originate from erythromyeloid progenitor cells in the developing embryonic yolk sac, and migrate into the embryonic CNS to differentiate into mature microglia. CNS microglia form a self-renewing cell population through proliferation and apoptosis throughout life of the individual [26, 27].

#### Characteristics and functions of microglia

**Fig. 1:**
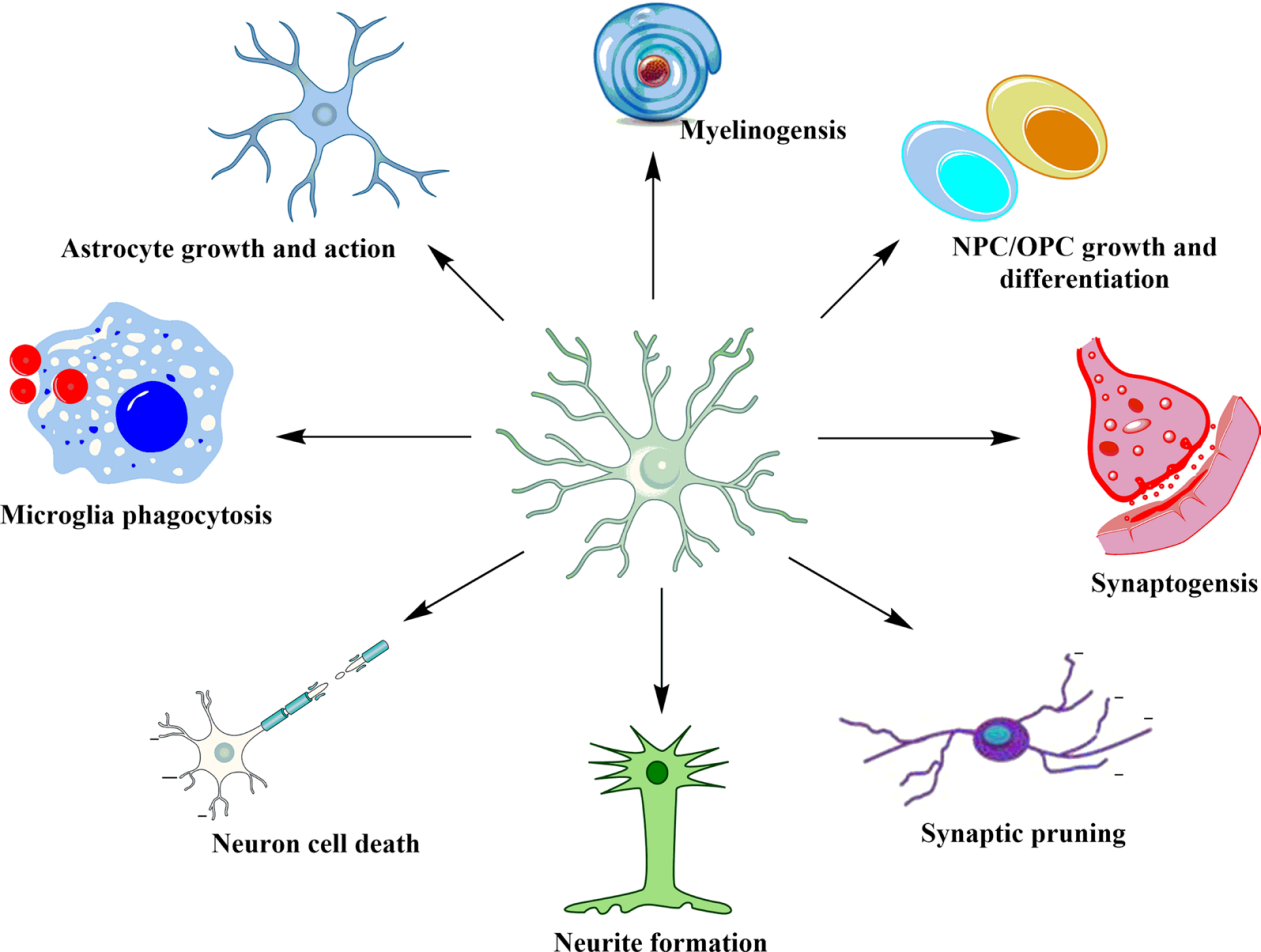
Multiple physiological roles of microglia in the CNS. Microglia are the center that maintain proper neuronal functioning of the CNS, including regulation of neuronal death, neurite formation, synaptic pruning, and synaptogenesis. In addition, microglia specify neural progenitor cell (NPC) and oligodendrocyte progenitor cell (OPC) growth and differentiation, facilitate myelination, and induce astrocyte activation. Lastly, microglia exhibit a phagocytic role concerning misfolded proteins and cellular debris [27]

As CNS immune effector cells, microglia are similar to peripheral macrophages with respect to their morphology and functions [28]. Under normal conditions, neurons serve a regulatory role in the CNS. Microglia provide protection and nutritional support to neurons, influence neuronal homeostasis, regulate synaptogenesis, and activate astrocytes (Fig. 1) [29, 30]. Resting microglia are characterized by a small soma with multiple symmetrically distributed protrusions that protrude at a rate of 0.4–3.8 μm/min to maintain either an extended or contracted state to sense changes in surrounding environment. Microglia are maintained in a relatively resting state in part by signals from neurons and astrocytes [31]. In response to challenges, such as tissue injury, pathogens, or other pathological processes, microglia quickly respond to the homeostatic imbalance and undergo considerable morphological transformation to provide defense mechanisms [32]. Specifically, the processes retract, the soma enlarges, and the ramification of the distal branches decreases. Activated microglia undergo additional morphological change and present a characteristic “amoeba-like” shape. The number of transformed microglia increases, and they migrate to the injured site at a rate of 1–2 μm/min for tissue repair [33, 34]. The activated microglia gradually shift from providing nutritional support and repairing neurons to neuronal dysfunction. These changes in function may be more persistent after continuous exposure to certain stimuli. This response further recruits peripheral innate immune cells (e.g., macrophages) and adaptive immune cells (e.g., B cells) to cross the blood–brain barrier and eventually lead to cognitive and emotional disorders [35–37].

#### The polarization of microglia

**Fig. 2:**
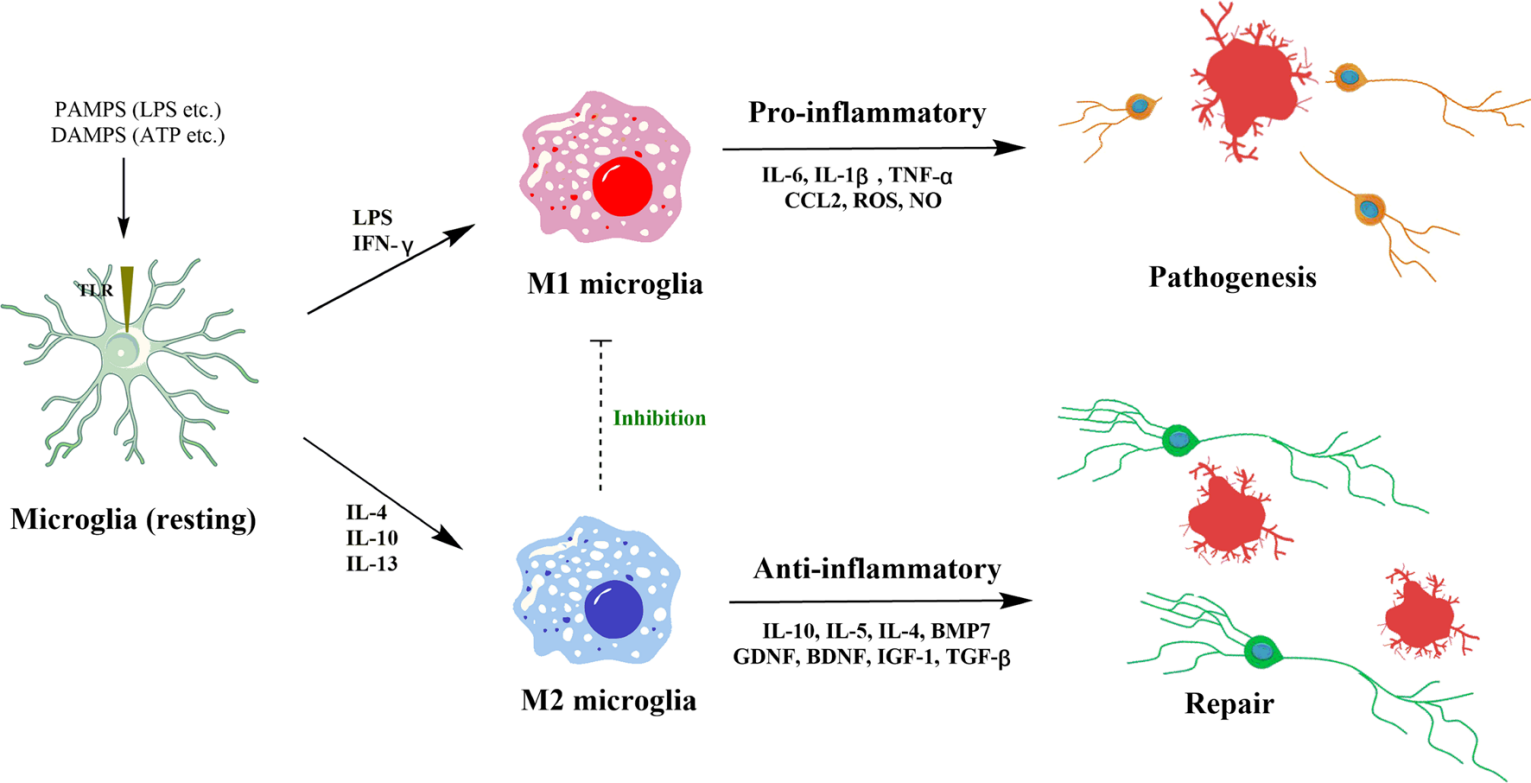
Microglial M1/M2 polarization and their regulatory functions in the CNS. Resting microglia are stimulated with pathogen-associated molecular patterns (PAMPs) or danger-associated molecular patterns (DAMPs) via toll-like receptors (TLRs). In the presence of lipopolysaccharide (LPS) and interferon-γ (IFN-γ), microglia polarize to the M1 phenotype and produce pro-inflammatory mediators including interleukin-1β (IL-1β), IL-6, tumor necrosis factor-α (TNF-α), CCL2, reactive oxygen species (ROS), and NO. In contrast, IL-4, IL-13, or IL-10 cause alternative activation into M2 microglia that inhibit M1 functions by releasing anti-inflammatory cytokines and neurotrophic factors [40]

Microglia exist in a resting state under normal physiological conditions and carry out “immune surveillance” functions [38, 39]. When CNS damage or infection occurs, microglia can be broadly divided into the classic activation (M1) or alternative activation (M2) phenotype. M1 microglia result in pro-inflammatory cytokine release and increased expression of several differentiation marker 86 and 16/32 (CD86, CD16/32) and inducible nitric oxide synthase (iNOS). M2 microglia are inclined to express anti-inflammatory cytokines, arginase-1 (Arg1), transforming growth factor-β1 (TGF-β1), CD206, and chitinase-3-like-3 [22]. M1 microglia remove apoptotic cells, pathogens, and inhibit normal neuron growth, which adversely affects synaptic transmission. M2 microglia promote phagocytosis of cell fragments and misfolded proteins, tissue repair, and support neuron survival. Intriguingly, M2 microglia are driven by the coordinated regulation of multiple anti-inflammatory mediators and against M1-induced inflammation that ultimately achieve immune suppression and neuronal protection (Fig. 2) [40].

However, the status of microglia may include a range of different but overlapping functional phenotypes that are in response to changes in their local environment. Novel single-cell technologies, such as single-cell RNA sequencing and cytometry by time-of-flight mass spectrometry, have emerged as superior methods to characterize immune cell types and states, the transition from normal to disease, and respond to therapy. For instance, microglia were isolated from LPS-injected mice that showed downregulation of homeostatic markers (e.g., Tmem119, Mef2c, P2ry13, P2ry12, and Siglech) and upregulation of inflammatory genes (e.g., Ccl2, Gpr84, and Nfkbia) by single-cell RNA sequencing and multicolor flow cytometry [41]. In the brains of a transgenic mouse model of Alzheimer’s disease (AD), a novel type of microglia (disease-associated microglia, DAM) was identified using single-cell analysis. Researchers further discovered that Trem2 is necessary for transformation from DAM stage-1 to DAM stage-2 [42]. With the development of unbiased and high-throughput analytical methods, it is possible to comprehensively characterize the spatial and temporal heterogeneity of microglia during CNS development and disease [43, 44]. Based on current research of microglial phenotypes, the classification using M1 and M2 subtypes is oversimplified and not universally accepted. We will use “pro-inflammatory” or “anti-inflammatory” microglia in subsequent descriptions to highlight the contribution of microglia in depression.

### Microglia in depression

#### Clinical evidence

A growing body of research has shown that damage to the typical structure and function of microglia in the developing and adult brain is associated with the etiology of depressive disorders. Changes in microglia in different brain regions, including the PFC, HIP, anterior cingulate cortex (ACC), and amygdala, are involved in the development of depression. Previous studies have shown that considerable microglial activation occurs in the PFC and ACC during severe episodes of MDD. Microglial activation in ACC also is positively correlated with the severity of the depressive episode [45]. Positron emission tomography (PET) scans have shown that microglia are increased in ACC during episodes of MDD [46]. At the same time, the TSPO levels (a marker of microglial activation) were increased in MDD patients [47]. A cross-sectional study using ^18^F-FEPPA PET showed a strong relationship between the total distribution volume of TSPO and the duration of untreated MDD, total illness duration, and antidepressant exposure [48]. Correspondingly, in an autopsy study of patients with MDD, the concentrations of quinolinic acid (QUIN) produced by microglia were increased in the subgenual ACC and anterior midcingulate cortex of suicide victims [49]. In the dorsal ACC white matter from depressed suicide patients, microglial density was increased significantly, as identified by elevated gene expressions of ionized calcium-binding adapter molecule 1 (Iba-1), CD45, and monocyte chemoattractant protein-1 [50]. Another study reported similar results that were obtained in the prefrontal white matter [51]. Nevertheless, the role of microglia-mediated inflammation in the occurrence of depression is still controversial. Additional evidence demonstrated that microglia decrease in depression. In subjects with familial MDD, glia cells were clearly reduced while the number of neurons remained unchanged [52]. Brain imaging revealed cortical atrophy in the subgenual part of Brodmann’s area 24 [52]. A similar reduction in glial density was also observed in the orbital cortex, ACC, and dorsolateral PFC, based on a laminar analysis [53–55]. In addition, the numbers of glial cells were reduced, especially on the left side of the amygdala in MDD patients, when assessed using stereological methods [56]. However, no reduction in the numbers of glia was found in area 3b of the somatosensory cortex in patients with depression, suggesting that glial reduction in mood disorders is limited to specific brain structures [57].

In addition to depression disorder, microglial activation has been reported in other mental disorders such as anxiety [58], schizophrenia [59], and autism spectrum disorders [60, 61]. Anxiety disorders often emerge early in life and are associated with other diseases. Clinical reports on microglia and anxiety disorders remains extremely limited, except in animal models of depression [58]. A PET imaging study demonstrated increased microglial activity in patients with schizophrenia and persons at ultra-high risk of psychosis [62]. In autism, increases in microglia were observed in cortical areas (fronto-insular and visual cortex) of the brain [63]. Conversely, several studies reported no significant microgliosis or changes in the expression of glial cell markers in schizophrenia and autism [60]. Therefore, there are different opinions concerning microglial activation in the development of these mental diseases, which may be related to assessments during different stages of disease onset or individual heterogeneity. The treatment of depression should be personalized based on the status of major depression and microglial function in individual patients. It also is worth noting that suicide has a high prevalence in the long-term course of mental diseases. Postmortem analysis has revealed that an elevated microglial density in schizophrenia and depressive patients is associated with suicide [59], indicating altered microglial activity might be critical in psychiatric disorders.

#### Preclinical evidence

**Fig. 3:**
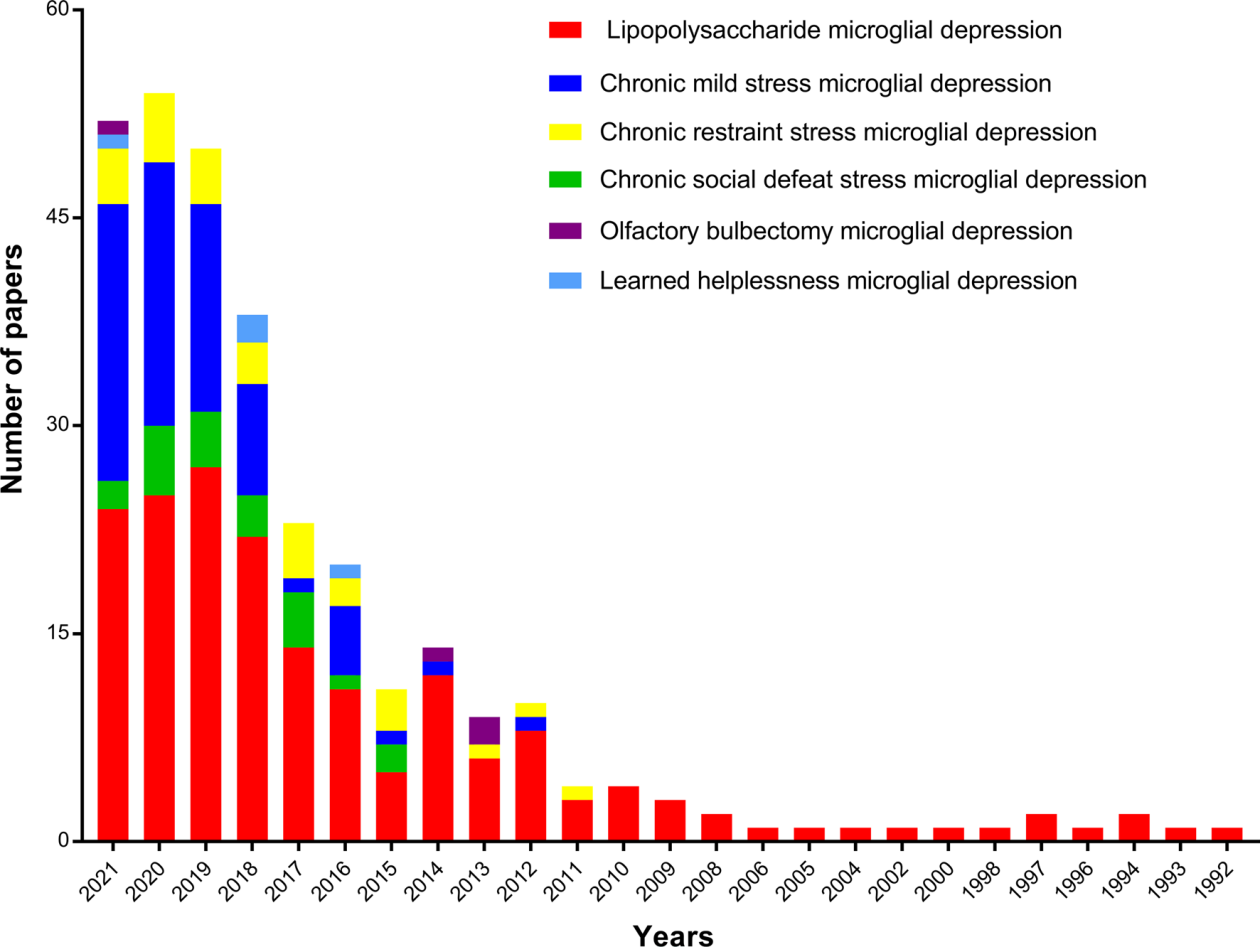
Annual changes in the number of published papers focused on animal models of depression and microglia

Considerable research has focused on the relationship between MDD and microglia in various animal models. Sustained microglial activation that exhibited high levels of pro-inflammatory cytokines has been observed in different brain regions [64–68], while inhibition of microglia alleviated depressive-type behaviors [69]. In this review, we illustrated the effects of microglial activation in animal models of depression, including acute/chronic stress and rodent pharmacological models. The search themes that were used included “LPS, chronic mild stress (CMS), chronic social defeat stress (CSDS), chronic restraint stress (CRS), olfactory bulbectomy (OBX), or learned helplessness (LH) combined with microglia and depression, respectively. The results indicated that microglia involved in nearly all models mentioned above. Among them, microglia were most evident in the models associated with LPS, CMS, and CRS, as well as CSDS-induced neuroinflammation, as shown in Fig. 3.

In a mouse model of acute depression, intraperitoneal injection of LPS activated the nod-like receptor pyrin containing 3 (NLRP3) expression and IL-1β cleavage in the HIP. Immunofluorescence staining showed that NLRP3 was primarily expressed in Iba-1 positive microglia when compared with control mice [70]. In BV2 microglial cells, LPS exposure induced an imbalance between the pro-inflammatory and anti-inflammatory microglial phenotype [71], activated TLR4/nuclear factor-kappa B (NF-κB) pathway, and downregulated TREM2 expression [72]. In the HIP and cortex of CMS-exposed mice, immunofluorescence staining revealed that the activated microglia (Iba-1 positive cells), as well as increased pro-inflammatory microglial markers (IL-1β, TNF-ɑ, IL-6, INF-γ, and iNOS) and decreased anti-inflammatory markers (Ym1, IL-4, IL-10, and Arg-1), suggesting a transformation of the microglial phenotype [73]. The CSDS paradigm also produces mood alterations and microglial activation, as well as ROS elevation. In addition, depletion of microglia using PLX5622 protects against behavioral abnormalities in the light–dark (LD) and social interaction (SI) tests [74]. These studies provide evidence that depressive-like behaviors and inflammation are present in chronic stress and pharmacological rodent models, which might be associated with persistent interference in microglia-related signaling. Other animal models that demonstrate an association between depression and microglia are shown in the Additional file 1: Table S1.

Since 1992, numerous studies have verified the concept that CNS microglia-mediated inflammation may contribute to depression and is closely related to regional selectivity and disease severity. Even though a strong correlation between microglia and depression has been observed in patients and animal models, determining whether microglial abnormalities actually play a significant role in depression remains challenging.

### The role of microglia in the pathogenesis of depression

#### Microglia–neuron communication in depression

Microglia–neuron communication functions bi-directionally. Microglia impart considerable influence on numerous aspects of neuronal function. Similarly, neurons regulate microglial functions mainly through soluble factors such as chemokines, cytokines, and neurotransmitters. Among these factors, CX3CL1 and CD200 are primarily expressed by neurons, and their receptors, CX3CR1 and CD200R, are expressed on microglia [75]. Dysfunctional interactions between neurons and microglia are critical factors in severe neurological disorders, including depression, schizophrenia, and AD.

The communication between CX3CL1 and CX3CR1 contributes to the ability of microglia to maintain functional stability. Depending on the degree of brain damage, CX3CL1 leads to increased microglia pro-inflammation or maintenance of microglia in a quiescence state [76, 77]. In a LPS-induced depression model, the decreased expression of CX3CL1 and microglial activation were observed in the HIP [78]. The serum levels of CX3CL1 in patients with moderate–severe depression were higher than in the control group [79]. A similar observation was made for the plasma levels of CX3CL1 from MDD patients with co-morbid cocaine addiction [80]. Cx3cr1-deficient mice displayed transient microglial reductions during the early postnatal period and subsequent defects in synaptic pruning. Defective synaptic pruning has been associated with less effective synaptic transmission and decreased neural circuit formation, and social interaction, as well as increased repetitive behavior, which also have been observed in several neuropsychiatric disorders. These findings suggest that microglia-mediated disruption of synaptic pruning could be associated with neurodevelopmental and neuropsychiatric disorders [81]. More importantly, previous studies have suggested that hyper-ramified microglia (process branching and/or soma enlargement) is connected to depressive-like behavior in rodents. The CX3CR1-deficient mice showed definite resistance to repetitive swim stress-induced depressive-like behavior and microglia hyper-ramification changes compared to wild-type mice [82]. Also, CX3CR1-deficient mice also demonstrated the impairment in long-term potentiation (LTP). Treatment with an IL-1β receptor antagonist significantly reversed the cognitive function and synaptic plasticity impairments observed in CX3CR1-deficient mice [83]. The prenatal stress produced behavioral disturbances in anxiety and depressive behavior in adult offspring of rats. The underlying mechanism may be related to the upregulation of CXCL12 and its receptor, as well as decreased CX3CL1-CX3CR1 expression in the HIP and frontal cortices, whereas exogenous CX3CL1 application alleviated the observed changes [84, 85]. Furthermore, CX3CR1 deficiency has been shown to impair neuron–microglia responsiveness to chronic stress [86]. Treatment with antidepressants such as fluoxetine, venlafaxine, or tianeptine normalized these behavioral and biochemical alterations [84].

Recently, CD200-CD200R has been shown to be related to the pathogenesis of depression through animal models studies that focused on using different stress-inducing protocols. For instance, exposure to inescapable tail shock resulted in reduced CD200R level in the HIP, basolateral (BLA), and central nucleus of the amygdale [87]. Similar observations have been reported in male and female rats [88]. However, a paradoxical observation indicated that unavoidable foot shocks in rats reduced the transcription level of CD200R in the hypothalamus, but not the HIP [89]. These discrepancies between published reports concerning the HIP might be attributed to the use of different protocols by the two research groups. In an IFN-α-induced model of depression, vulnerable mice displayed increased levels of MHC-II, CD86, and CD200R. These mice displayed depressive-like behaviors characterized by increased immobility time in the forced swimming test (FST) and tail suspension test (TST), as well as decreased explorative behavior in the novel object exploration test [90].

### Microglia and neurogenesis in depression

During brain development, microglia regulate synaptic transmission, prune neuronal synapses, and facilitate the formation of neural circuits. Once homeostasis is disturbed, microglia are converted into an active state and release pro-inflammatory cytokines, chemokines, and reactive oxidants. The pro-inflammatory microglia are involved with the upregulation of pro-inflammatory mediators, which is usually considered a harmful event. Whereas anti-inflammatory microglia display protective effects in neuronal survival and adult neurogenesis [91].

Activation of microglia is a critical mechanism in neurogenesis inhibition in the presence of inflammation and stress [9]. Hippocampal neurogenesis is a complex neurobiological process involving the generation and functional integration of newborn cells into brain neural circuits [92]. Several studies have demonstrated that stress strongly suppresses adult hippocampal neurogenesis. Both acute and chronic stress have been reported to reduce adult neurogenesis by decreasing neuroprogenitor proliferation and newborn cell survival [93–95], and also impair newborn neuron maturation [96, 97]. LPS infusion via a cannula for 4 weeks reduced the survival of new neurons in the subgranular zone/granule cell layer (SGZ/GCL) of the HIP, while 6 days of intracortical LPS infusion in rats did not affect the proliferation of new cells [98]. In addition, LPS-induced neuroinflammation suppressed proliferation and differentiation of neural stem cells in the dentate gyrus (DG) of the HIP, which was indicated by the decreased number of BrdU-, DCX- and NeuN-positive cells [99]. In an 8-week CMS-induced depression paradigm, C57BL/6 mice exhibited an increase in the number of Iba-1 positive cells and levels of IL-1β, IL-6, and TNF-α [100]. Another report on the CMS showed that an imbalance of peripheral inflammation markers and decreased CX3CL1/CX3CR1 immunoreactivity were associated with reduced numbers of BrdU/Ki-67/DCX^+^ (nascent, proliferating and DCX-associated) cells in the DG of the HIP [101]. Furthermore, in a graded study of the CMS for 5, 6, or 7 weeks, the investigators found that the pro- or anti-inflammatory microglial changes in the cortex and HIP, as well as BrdU/DCX^+^ cells, decreased in the DG [102]. Similar results related to the relationship between depressive-like behaviors and changes in microglia and neurogenesis have been reported in psychosocial stress models, including maternal separation [103], maternal sleep deprivation [104], chronic water-immersion restraint stress (CWIRS) [105], and CSDS [106, 107]. On the other hand, IL-4 and IL-10 induce alternative activation of anti-inflammatory factors that play a critical neuroprotective function in tissue remodeling and nerve regeneration. Treatment with IL-4 markedly inhibited IL-1β-caused depressive behavior by regulating glial activation and neurotransmitter levels in the HIP [108]. IL-4 stimulation increased the expression of insulin-like growth factor-1 in microglia, which has been reported to promote neurogenesis [109, 110]. Recent research demonstrated that IL-4-driven Arg1^+^ microglia modulate stress resilience through brain-derived neurotrophic factor (BDNF)-dependent neurogenesis in CMS mice [111]. IL-10-stimulated microglia enhanced the proliferation of NPCs but did not affect neuronal differentiation. IL-10-secreting microglia supported neuronal differentiation and the survival of newly formed cells in vitro [112]. CX3CR1^CreER^IL-10 knockout mice exhibited depression- and anxiety-like behaviors, along with decreased NR2B (N-methyl-D-aspartate receptor (NMDAR) subunit) and synaptophysin (SYP) levels in the mPFC, and increased NR2B and postsynaptic densitin-95 (PSD95) levels in the amygdala [113]. Furthermore, hippocampal neurogenesis was reduced in CX3CR1-deficient mice, and antagonizing CX3CR1 resulted in increased hippocampal IL-1β level and decreased neurogenesis in young rats [114]. Minocycline, a microglial inhibitor, reversed the pathogenic phagocytic potential of neurotoxic microglia, and reduced the negative phenotypes associated with reduced neurogenesis in depression models induced by chronic stress and LPS [115]. Therefore, these data suggest that microglia are correlated with reduced neurogenesis induced by exposure to stress and, in turn, may be responsible for the development of depressive-like behaviors.

### Microglia-mediated activation of NLRP3 in depression

The NLRP3 inflammasome is a multi-molecule complex containing cytosolic NLRP3, adaptor protein ASC, and pro-caspase-1 precursor. NLRP3 plays a vital role in production of pro-inflammatory cytokines during the stress process [116]. The NLRP3 inflammasome is currently considered to be an essential molecular platform for regulating pro-inflammatory cytokines release. NF-κB nuclear translocation is an indispensable event in the initiation of NLRP3 activation, which further confirmed that NF-κB nuclear translocation might play a role in assembling the NLRP3 inflammasome. When NLRP3 is activated by repetitive stress, it modulates caspase-1 activation, which, in turn, promotes IL-1β and IL-18 maturation in microglia, where excessive secretion of cytokines contributes to the development and progression of MDD [117–119].

NLRP3 inflammasome activation has even been observed in depressive patients and numerous animal models of depression. Previous studies have revealed that the NLRP3 inflammasome was activated in blood cells from MDD patients, and the levels of IL-1β and IL-18 were increased in serum. The increase in pro-inflammatory factors was positively correlated with Beck Depression Inventory scores [120], suggesting that NLRP3 might have a critical role in mediating the development of depression [121]. In another experimental study, a sustained CMS procedure (12 weeks) enhanced the levels of IL-1, NLRP3, ASC, TLR2, NF-κB, p-IKKα, and IKKβ in rat PFC, while these alterations were reversed with fluoxetine [122]. Immunofluorescent analysis has confirmed that the NLRP3 inflammasome was primarily activated only in microglia in the PFC (Iba-1/NeuN^+^ cells) [122]. Using NLRP3 inflammasome inhibitors AC-YVAD-CMK and VX-765 significantly improved depressive-like behavior, as shown by improvements in sucrose intake in the sucrose preference test (SPT) and immobility time in the FST of CMS mice [94, 123]. Moreover, activation of P2X7R and NLRP3 inflammasome-associated proteins in hippocampal microglia could mediate depressive-like behaviors, which provide new therapeutic targets for depression [124]. In a LPS-induced acute depression mouse model, NLRP3, ASC, and caspase-1 mRNA expressions were remarkably increased compared to the control group [125]. In addition, IL-1β secretion was closely controlled by the NLRP3 inflammasome, which plays an important role in the pathogenesis of depression [108]. Minocycline has an acute antidepressant role in CRS, CUMS, and LH models by inhibiting microglia and NLRP3 activation [126–129]. Fluoxetine conferred an antidepressant effect in part by inhibiting NLRP3 inflammasome activation [130]. Clomipramine reversed LPS-induced increases in IL-1β, IL-6, TNF-α, and NLRP3 gene expressions in vivo, as well as in vitro in BV2 cells [131]. Electro-acupuncture treatment for 4 weeks markedly reversed CMS-induced increases in NLRP3 inflammasome-associated components (NLRP3, ASC, and caspase-1) and the expressions of mature IL-1β, as well as IL-18, TNF-α, IL-6, P2X7 receptor, and Iba-1 [132]. Iptakalim negatively regulates NLRP3 expression and, in turn, affects microglia-mediated neuroinflammation by inhibiting the activation of NLRP3/caspase-1 axis in the HIP of CMS mice [94]. These findings suggest that microglial NLRP3 activation is a central mediator involved in depressive-like behaviors in animal models and MDD patients.

### Microglia-mediated kynurenine pathway in depression

Chronic stress stimulation or inflammation may contribute to tryptophan (TRP) metabolism associated with the kynurenine pathway (KP). The generation of neuroactive kynurenine metabolites leads to subsequent depressive-like symptoms [133]. KP metabolizes tryptophan into several bioactive metabolites in the brain, including QUIN (an NMDAR agonist) and kynurenic acid (KYNA, an α7-AChR and NMDAR antagonist). KYNA is primarily formed in astrocytes [134], which exhibit a neuroprotective function via their ability to eliminate glutamate spillover. QUIN is produced mainly by microglia [135] and has a strong excitotoxic role through enhancing NMDAR activation [136]. Microglia regulate KP balance by preferentially producing oxidative metabolites, including QUIN. Other metabolites of KP, including 3-hydroxykynurenine (3-HK) and anthralinic acid, do not directly affect neuronal activity but are involved in complicated pro-oxidation and anti-oxidation processes [137].

Recent studies have found that microglial QUIN expression was increased in the subgenual and supracallosal regions of the ACC in post-mortem brains of suicide patients with severe depression. In contrast, additional research revealed decreased QUIN in left CA1 or right CA2/3 areas of the HIP in uni- and bipolar depression patients [138]. Moreover, a meta-analysis revealed decreased KYNA and kynurenine (KYN) levels in patients with depression and increased QUIN level in antidepressant-free patients [139]. Similar results have been reported concerning the increase of QUIN in peripartum depression and adolescent MDD [140, 141]. In addition to clinical data, enhanced levels of 3-HK and QUIN have been observed in several models of depression [142, 143]. LPS and other infectious agents, including viruses, upregulate the release of inflammatory cytokines by binding to TLRs, which in turn directly and indirectly induce KP metabolism via pro-inflammatory cytokines [144]. Ketamine treatment could reduce the LPS-induced depressive-like alterations observed in the novelty suppressed feeding test (NSFT) and splash test (ST) [145]. In a genetic animal model, the level of KYNA was reduced in the PFC of Flinders sensitive line rats compared with Flinders resistant line control rats [146]. In addition, CMS contributed to QUIN production and its release from microglia in the HIP. QUIN resulted in the elevation of Glu level via NMDARs and mGluR1, as well as the increased expression of NR2B and mGluR1, which lead to depressive-like symptoms [147]. CRS exposure induced depressive-like behavior in C57BL/6 J mice, which was attributed partially to disruption of the neuroprotective/neurotoxic balance of the kynurenine metabolic pathway in the gut and brain [148]. Enzyme indoleamine 2,3-dioxygenase 1 (IDO1) is considered to be a rate-limiting enzyme for tryptophan metabolism in KP [149]. Treatment with the IDO inhibitor, 1-methyl-tryptophan, partially prevented CRS-induced depression- and anxiety-like changes [148]. The chronic forced swim test and tail suspension test in mice enhanced KYN/TRP and reduced the 5-HT/TRP ratio, which indicated activation of IDO1 [150]. In the poststroke depression (PSD) mouse model, 3-hydroxyanthranilate 3,4-dioxygenase (HAAO), QUIN, IDO1, Iba-1, and ROS were remarkably increased in the nucleus accumbens (NAc), HIP, and hypothalamus. At the same time, treatment with aripiprazole ameliorated the abnormal behaviors in PSD mice that were accompanied by decreased levels of IDO1, HAAO, QUIN, and ROS [151]. IDOInh is a specific inhibitor of IDO1 and has been shown to reverse fear learning and memory in CSDS-induced depressed mice by decreasing KYN and 3-HK levels in the blood and brain [152]. Consequently, it has been proposed that kynurenine metabolites directly interact with microglial activity, which provides a reliable target for investigating the mechanisms underlying antidepressant drugs.

### Effects of antidepressants on microglia

Currently, numerous reports using different experimental models have indicated that antidepressants, including clinical and plant-based drugs, exert their antidepressant effects, in part, by regulating microglial phenotypes. Regulation of microglia has been proposed as a potentially effective therapeutic strategy in chronic inflammatory diseases [29, 30]. In this section, we summarized the current knowledge of antidepressants (clinical medicine and natural products) that act against microglial dysfunction in stress-induced depression models (Tables 1 and 2).

**Table 1:** Traditional antidepressants regulate the microglia-mediated neuroinflammation in animal models of depression

Treatment	Animal models	Behavioral test	Analyzed regions	Microglia	Pro-inflammation	Anti-inflammation
Fluoxetine [154]	CSDS	OFT, EPM, SIT	Serum	—	ELISA: TNF-α ↓, HMGB1 ↑	—
			Hip	Iba-1(–)	mRNA:TNF-α, IL-1β, RAGE, TLR4 ↑ Proteins: T-HMGB1, T-p65, IκB ↑, TLR4 ↓	mRNA: Arg-1, CD206 ↑
Fluoxetine [170]	CMS	Nest-building test, Dexamethasone suppression test	Cortical	CD11 b/P2X7R^+^↓	—	—
			Amygdala	CD11 b/P2X7R^+^↓	—	—
			HIP	CD11 b/P2X7R^+^↓	—	—
Fluoxetine [105]	CWIRS	SPT, FST, TST	HIP	Proteins: CD68, Iba-1 ↓	Proteins: IL-1β, TNF-α, iNOS ↓	—
Fluoxetine [155]	LPS	SPT, FST	HIP	Iba-1/COX-2^+^↓ in DG	ELISA: IL-1β, IL-6, TNF-α Proteins: TLR4, NLRP3 ↓	—
Citalopram [101]	CMS	SPT, FST	HIP	Iba-1^+^↑; CX3CL1^+^↑, CX3CR1^+^↑ in DG	—	—
Escitalopram [16]	CMS	OFT, SPT, TST, FST, NSFT	HIP	—	Proteins: IL-6, IL-1β ↓	IL-10 (–)
			Cerebral cortex	—	Protein: TNF-α ↓	IL-10 (–)
Imipramine [107]	CSDS	SIT, SPT, TST, FST	Serum	—	ELISA: IL-1β, IL-6, TNF-α ↓	—
			HIP	Iba-1^+^↓ in DG	mRNA: IL-1β, IL-6, TNF-α ↓ Proteins: p-p65/p65, cleved-Caspase-3, ac-NF-κB ↓	—
Sertraline [171]	CMS	SPT, TST, FST	PFC	Iba-1/HMGB1^+^↓	Proteins: TNF-α, IL-1β ↓	—
			Serum	—	ELISA: TNF-α, IL-1β, NO ↓	—
Sertraline [172]	CMS	TST, FST	Brain	Protein: Iba-1 ↓	Proteins: TNF-α, iNOS, IL-1β, p-p65-NF-κB, p65-NF-κB, p-IκB-α ↓	—
			Serum	—	ELISA: TNF-α, IL-1β ↓	—
Vortioxetine [17]	LPS	OFT, SPT, NORT	Dorsal HIP	mRNA: CD14, CD86 ↑	mRNA: TNF-α, IL-6, IDO1 ↑	mRNA: IL-4, TGF-β1 ↑
			Ventral hippocampus (vHIP)	mRNA: CD14, CD86, CD11b ↑	mRNA: IL-1β, IL-6, IDO1 ↑	mRNA: IL-4, IL-1Rα ↑
Imipramine [173]	Repeated social defeat	OFT, social avoidance test	Plasma	—	ELISA: IL-6, CORT ↓	—
			Brain	Proteins: CD45, CD11b ↓	—	—
Imipramine [156]	LH	—	Hilus	Iba-1^+^↓	—	—
Clomipramine [131]	LPS	TST, FST	HIP	Iba-1^+^↓	mRNA: IL-1β, TNF-α, IL-6 ↓	—
Agomelatine [174]	LPS	—	vHIP	mRNA: CD11b, CX3CL1, CX3CR1 ↓, CD68 ↑	—	—
Melation [70]	LPS	TST, FST	HIP	Iba-1/NLRP3^+^↓	Proteins: pro-IL-1β, IL-1β, NLRP3, caspase-1	—
SRT2104 [175]	CMS	OFT, SPT, TST, FST	HIP	Iba-1^+^, CD11b^+^ CD45^low+^ (–)	mRNA: IL-6, IL-1β, iNOS ↓ CD11b^+^ MHCII^+^↓	mRNA: IL-10, TGF-β, Arg-1 ↑ CD11b^+^CD206^+^↑
Pioglitazone [169]	CMS	OFT, SPT, TST, FST	HIP	Iba-1^+^↓	mRNA: IL-1β, IL-6, TNF-α, iNOS, CCL2 ↓	mRNA: Ym1, Arg1, IL-4, IL-10, TGF-β ↑
Clemastine [176]	CMS	SPT, TST, FST	Serum	—	ELISA: IL-1β, TNF-α ↓	—
			HIP	Protein: Iba-1 ↓	Proteins: IL-1β, TNF-α, iNOS ↓	Protein: Arg-1 ↑
Minocycline [163]	OBX + spinal nerve ligation	OFT	PFC	—	mRNA: IL-1β, IL-6 ↑	mRNA: MRC2, IL-10 ↑
Minocycline [14]	CMS	SPT, FST, EMP	Serum	—	CORT ↓	—
			HIP	mRNA: CD11b, IFN-γ/IL-4, IFN-γ/IL-10 ↓	mRNA: IFN-γ, TNF-α, IL-1β, IL-17 ↓ Protein: CD11b ↓	mRNA: TGF-β1, IL-4, IL-10, IL-13 ↑
Memantine [177]	OBX	Emotional behavior, TST, FST	HIP	Iba-1^+^↓	Proteins: p-IkBα, p-p65-NF-κB, TNF-α, IL-6 ↓	—
Iptakalim [94]	CMS	SPT, TST, FST	Serum	—	ELISA: IL-1β ↓	—
			HIP	MAC-1^+^↓ in DG	mRNA: TNF-α, IL-6 Proteins: p65, IL-1β, NLRP3, caspase-1 ↓	mRNA: IL-10 ↑
Simvastatin [178]	LPS or CMS	SPT, FST, NSFT	HIP	Iba-1^+^↓	Proteins: p65-NF-κB, IL-1β, TNF-α, IL-6 ↓	—
ONO-2952 [179]	CSDS	OFT, EMP, TST, FST	NAc	—	Proteins: TNF-α, IL-6 ↓	—
			BLA	DHE/Iba1^+^↓	Proteins: IL-1β, IL-6, IL-12 ↓	—
			PFC	—	Proteins: IL-1β, IL-12 ↓	—
			vHip	—	Protein: IL-12 ↓	—
Iptakalim [180]	CRS	OFT, FST	Hypothalamus	TNF-α/CD11b^+^↓	mRNA: TNF-α, TLR4, IL-1β ↓	—
Caffeine [105]	CWIRS	SPT, FST, TST	HIP	Protein: Iba-1 ↓ CD68^+^↓	Proteins: CD68, iNOS, TNF-α, IL-1β ↓	—
Apelin-13 [181]	CWIRS	OFT, SPT, TST, FST	HIP	Iba-1^+^↑, Iba-1/iNOS^+^↑, Iba-1/Arg-1^+^↓ in CA1	Proteins: IL-1β, IL-6, iNOS ↓	Proteins: IL-1β, IL-6, Arg-1 ↑
Melatonin [101]	CMS	SPT, FST	HIP	Iba-1^+^, CX3CL1^+^, CX3CR1^+^↑ in DG	—	—
ω-3 polyunsaturated fatty acids [182]	Ovariectomized	SPT, FST, NSFT, TST	HIP	Iba-1^+^↓ in DG	mRNA: IL-1β, IL-6, TNF-α, CD68 ↓ Proteins: p-p65, IκB, iNOS ↓	mRNA: IL-4, IL-10, CD206, Arg-1 ↑ Protein: Arg-1 ↑
Fingolimod [183]	CMS	OFT, SPT, FST, WMW	HIP	mRNA: Iba-1 ↑	ELISA: IL-1β, IL-6, TNF-α ↓ mRNA: NLRP3, ACS, caspase-1, iNOS, CD16 ↓ Proteins: iNOS, CD16, NLRP3, ACS, caspase-1 ↓	ELISA: IL-10 ↑ mRNA: Arg-1, CD206 ↑ Proteins: Arg-1, CD206 ↑

↑upregulated; ↓downregulated; (–) no significant difference;—no explicit data

**Table 2:** Plant-derived natural compounds and formulations that regulate microglia-mediated neuroinflammation in animal models of depression

Treatment	Animal models	Behavioral test	Analyzed regions	Microglia	Pro-inflammation	Anti-inflammation
Crocin [226]	LPS	OFT, FST, TST, FST	HIP	—	ELISA: IL-1β, IL-18, TNF-α ↓ Proteins: CD16/32, iNOS, p-65-NF-κB ↓	Protein: CD206 ↑
Baicalin [186]	CMS	OFT, FST, TST, FST	HIP	—	Proteins: IL-1β, IL-6, TNF-α, TLR4 ↓	—
Catalpol [205]	CMS	OFT, EPM, FST	HIP	Iba-1^+^↓	mRNA: IL-1β, TNF-α, iNOS, IL-6, CD206 ↓ Proteins: NLRP3, cleaved caspase-1, IL-1β ↓ ROS ↓	mRNA: CD206 ↑
Ganoderic acid A [227]	MCAO + CMS	OFT, SPT	HIP	—	ELISA: TNF-α, IL-1β, IL-6 ↓ mRNA: TNF-α, IL-1β, IL-6, iNOS, CD68 ↓ Proteins: iNOS, CD68 ↓	ELISA: IL-10 ↑ mRNA: IL-10, CD206, Arg-1 ↑ Proteins: CD206, Arg-1 ↑
Ginsenoside Rb1 [73]	CMS	OFT, SPT, TST, FST	HIP	Iba-1^+^↓	mRNA: TNF-α, IL-1β ↓	mRNA: TGF-β, Arg-1 ↑
			Cortex	Iba-1^+^↓	mRNA: TNF-α, IL-1β ↓	mRNA: TGF-β, Arg-1 ↑
Salvianolic acid B [213]	CMS	SPT, TST, FST	HIP	Iba-1^+^(–)	ELISA: TNF-α ↓ mRNA: IL-1β, TNF-α ↓	ELISA: IL-10 ↑ mRNA: IL-10, TGF-β ↑
			Cortex	Iba-1^+^↓	ELISA: TNF-α ↓ mRNA: IL-1β, TNF-α ↓	ELISA: IL-10 ↑ mRNA: IL-10, TGF-β ↑
Curcumin [215]	GWI + CRS	OLT, NORT, NSFT	HIP	Iba-1^+^↓	—	—
Ginsenoside Rb1 [197]	CRS	OFT, TST, FST	HIP	Protein: Iba-1 ↓	Proteins: TNF-α, IL-1β ↓	—
Arctigenin [171]	CMS	SPT, TST, FST	Serum	—	ELISA: TNF-α, IL-1β, NO ↓	—
			PFC	Iba-1/HMGB1^+^↓ Protein: Iba-1 ↓	Proteins: TNF-α, IL-1β, IDO ↓	—
Astragalin [231]	CMS	SPT, TST, FST	HIP	Iba-1^+^↓	Proteins: Nuclear p65-NF-κB, NLRP3, cleaved caspase-1, cleaved IL-1β, cleaved gasdermin D ↓	—
Saikosaponin-d [190]	LPS	OFT, FST, TST, FST	HIP	Iba-1^+^↓ in CA1	Proteins: CD68, IL-1β, IL-6, TNF-α, HMGB1, TLR4, p65-NF-κB, p-IκB-α ↓	—
Asperosaponin VI [228]	LPS	OFT, FST	HIP	Iba-1^+^↓	mRNA: IL-1β, IL-6, TNF-α, iNOS ↓ Proteins: IL-1β, TNF-α, TLR4, p-NF-κB/NF-κB ↓	—
			PFC	Iba-1^+^, iNOS/ Iba-1^+^↓	mRNA: IL-1β, IL-6, TNF-α, iNOS ↓ Proteins: IL-1β, TNF-α, TLR4, p-NF-κB/NF-κB ↓	
5-*O*-Methylvisammioside [233]	LPS	OFT, FST, FST, EPM	HIP	—	Proteins: NF-κB, IκB-α ↓	—
Scutellarin [230]	LPS	OFT, SPT, FST	HIP	Iba-1^+^↓	Proteins: NLRP3, caspase-1, IL-1β ↓ ROS ↓	—
Ginsenoside Rg1 [200]	CMS	SPT, FST	vmPFC	Iba-1^+^↓	mRNA: IL-1β, IFN-γ, TNF-α ↓ ELISA: IL-1β, IFN-γ, TNF-α ↓	—
Ginsenoside Rg3 [201]	LPS	—	Cortex	Iba-1^+^↓	—	—
			Hypothalamus	Iba-1^+^↓	—	—
			Brain	iNOS^+^, COX-2^+^, Iba-1^+^↓	mRNA: IL-1β, IL-6, TNF-α	—
Magnolol [219]	CMS	OFT, SPT, FST, TST	HIP	Iba-1^+^↓ in DG; Iba-1/CD16/32^+^↓, Iba-1/CD206^+^↑	—	—
			Brain	—	ELISA: TNF-α, IL-1β, IL-6, IL-12 ↓	ELISA: IL-4, IL-10 ↑ mRNA: Arg1, Ym1, Fizz1, Klf4 ↑
Geniposide [208]	LPS	SPT, TST, FST, OFT	HIP	—	ELISA: TNF-α, IL-6 ↓ Proteins: CD86 ↓, CD206 ↑	—
			Serum	—	ELISA: TNF-α, IL-6 ↓	—
Arctiin [210]	CMS	TST, FST, OFT, SPT	PFC	Iba-1^+^/HMGB1^+^↓ Protein: Iba-1 ↓	Proteins: TNF-α, IL-1β, iNOS, HMGB1 ↓	—
			Serum	—	ELISA: TNF-α, IL-1β, iNOS ↓	
Ginkgolide B [234]	Depression in post myocardial infarction	OFT, SPT	Median raphe nucleus	—	ELISA: IL-1β ↓ mRNA: IL-1β ↓	—
			HIP	Iba-1^+^↓	ELISA: IL-1β ↓ mRNA: IL-1β ↓	—
			Cortex	—	ELISA: IL-1β ↓ mRNA: IL-1β ↓	—
Leonurine [235]	CMS	TST, FST, SPT	HIP	—	Proteins: IL-1β, IL-6, TNF-α, p-IKKβ/IKKβ, p-p65/p65 ↓	—
Hesperidin [232]	CMS	FST, SPT, OFT	PFC	Iba-1^+^↓	mRNA: NLRP3, caspase-1, ASC ↓ Proteins: NLRP3, caspase-1, ASC ↓ ELISA: IL-1β, IL-6, TNF-α ↓	—
20(*S*)-Protopanaxadiol [229]	CMS	SPT, TST, FST	HIP	Iba-1^+^↓	Proteins: iNOS, COX-2, acetylated p65 (ac-p65) ↓	—
			Cortex	Iba-1^+^↓	—	—
Ferulic acid [236]	CMS	SPT, FST	PFC	—	mRNA: IL-6, IL-1β, TNF-α, NF-κB, CD11b ↓ Proteins: NLRP3, caspase-1 ↓	—
Resveratrol [224]	LPS	OFT, TST, FST	HIP	Iba-1^+^↓ in DG-SGZ	—	—
(+)-Sesamin [69]	CMS	OFT, FST, TST, EMP	HIP	Protein: Iba-1 ↓	mRNA: COX-2, iNOS, IL-1β, TNF-α ↓	—
			Prelimbic cortex	Protein: Iba-1 ↓	mRNA: COX-2, iNOS, IL-1β, TNF-α ↓	—
Quercetin [187]	OBX	OFT, FST	HIP	—	ELISA: TNF-α, IL-6 ↓	—
			Cortex	—	ELISA: TNF-α, IL-6 ↓	—
Theaflavins [246]	LPS	TST	HIP	MIP-1α/CD11b^**+**^, TNF-α/CD11b^**+**^↓ in microglia	ELISA: TNF-α, IL-1β ↓	—
Epigallocatechin-3-gallate [99]	LPS	—	HIP	Iba-1^+^↓ in DG	mRNA: IL-1β, IL-6, TNF-α ↓ Proteins: TLR4, Rel A, pRel A ↓ ELISA: IL-1β, IL-6, TNF-α ↓	—
Gypenosides [192]	CMS	SPT, TST	HIP	Iba-1^+^↓ in DG	mRNA: IL-6 ↓ Proteins: IL-6, TNF-α ↓	—
*Kososan* [237]	CSDS	Social avoidance test	HIP	Iba-1^+^↓	—	—
*Aquilariae Lignum* ethanol extracts [238]	CRS	—	HIP	Iba-1^+^↓	Proteins: TNF-α, IL-1β, iNOS ↓	—
*Rosemary* Extracts [239]	CRS	OFT, TST, FST	Serum	—	ELISA: IL-1β, TNF-α ↓	—
			HIP	Protein: Iba-1 ↓	Proteins: IL-1β, TNF-α, p-p65-NF-κB ↓	—
*Radix Polygalae* extract [240]	CRS	OFT, SPT, NSFT	PFC	Iba-1^+^↓	mRNA: NLRP3, IL-1β, IL-6, IL-18, TNF-α ↓ Proteins: NLRP3, ASC, cleaved caspase-1	—
*Bangpungtongsung-san* [241]	Reserpine	OFT, TST, FST	HIP	—	mRNA: IL-1β, IL-6, TNF-α ↓	—
*XingnaoJieyu* decoction [242]	MCAO + CMS	OFT, FST	Cortex	Iba-1^+^↓	ELISA: TNF-α, IL-6, IL-1β ↓	—
			HIP	Iba-1^+^↓	ELISA: TNF-α, IL-6, IL-1β ↓	—
Ganoderma lucidum polysaccharides [243]	CSDS	OFT, SPT, TST, FST	HIP	Iba-1^+^↓	Proteins: IL-1β, TNF-α ↓	Proteins: IL-10, BDNF ↑
Water extract of *Armillaria mellea (Vahl)* P. Kumm [244]	CMS	OFT, FST	Cerebral	Protein: Iba-1 ↓	Proteins: IL-1β, TNF-α ↓	—
*Xiaoyaosan* [245]	CMS	SPT, OFT, FST, NSFT	HIP	Iba-1^+^↓	mRNA: COX-2 ↓	—
*Myelophil* [96]	CMS	OFT, TST, FST	HIP	Iba-1^+^↓ in CA1, DG, CA3	ELISA: IL-1β, TNF-α ↓ Proteins: NLRP3, ASC, pro-IL-1β, IL-1β ↓	—

↑upregulated; ↓downregulated; (–) no significant difference;—no explicit data

### Clinical antidepressive drugs

Clinical antidepressants, including selective serotonin reuptake inhibitors (SSRIs) and tricyclic antidepressants (TCAs), have exhibited effects on microglial activation and neuroinflammation [153]. Other compounds, including minocycline, melation, FCPR16, pioglitazone, iptakalim, and caffeine, also influence microglial polarization and depressive-like behavior [91], as shown in Table 1.

The SSRIs (fluoxetine, citalopram, and escitalopram) exhibit antidepressant roles in LPS, CMS, CSDS, and CWIRS models, as seen by alleviating nest-building deficits, reducing immobility time in the FST and TST, and increasing sucrose intake in the SPT (Table 1). These behavioral changes were accompanied by decreases in the microglial marker Iba-1 expression in different brain regions. In addition, pro-inflammatory factors levels (IL-1β, TNF-α, and NO) were downregulated, and anti-inflammatory factors levels (IL-4, IL-10, Arg-1, and TGF-β1) were upregulated [16, 101, 105, 154, 155]. Tricyclics, including clomipramine and imipramine, reversed depressive behaviors induced by LPS, LH, and CSDS by reducing the number of activated hippocampal microglia (Table 1). These traditional antidepressants promote the activation of anti-inflammatory microglia to release anti-inflammatory cytokines and neurotrophic factors, suggesting that reducing inflammation may be part of the function of clinical antidepressants [107, 131, 156]. Cytokines levels are strongly correlated with the efficacy of antidepressant treatment in patients. For example, higher levels of IL-6 and TNF-α are more commonly found in treatment-resistant patients than responders [157]. Nevertheless, some results revealed conflicting views that antidepressants might sometimes increase the inflammatory load in the brain. For instance, citalopram treatment elevated the levels of TNF-α and IFN-γ in the PFC, and these effects were inhibited by the anti-inflammatory agent ibuprofen [158]. Phenelzine, a monoamine oxidase inhibitor, has been shown to enhance the microglia-mediated immune responses by increasing the expressions of iNOS, TNF-α, and IL-6 in LPS-treated BV-2 cells. It has been verified that phenelzine also increased the levels of NO, TNF-α, and IL-6 via the NF-κB signaling pathway in LPS-activated primary microglia cells [159]. These differences may depend on regional brain specificity and heterogeneity among depression models and patients, but the conflicting causes and mechanisms need further study. Ketamine has exhibited potential antidepressant effects in vivo and in vitro by inhibiting microglia-mediated neuroinflammation [160]. Partial depletion of microglia with PLX3397 blocked the rapid and sustained antidepressant effects of (*R*)-ketamine (an isomer of ketamine), suggesting that antidepressant effects of ketamine may be partly attributed to microglial activation [161].

Minocycline is a potential agent for the treatment of depression [19]. In several studies, chronic minocycline treatment clearly alleviated depressive-like symptoms by inhibiting microglia and HPA axis hyperactivity. The powerful neuroprotective effects of minocycline were mainly mediated by modulating pro-inflammation and anti-inflammation balance in the PFC and HIP, as well as upregulating the BDNF-mediated synaptic plasticity in stress models [14, 162–164]. Meanwhile, CMS-induced changes in behavior, hippocampal LTP, CD11b expression, NLRP3 inflammasome, BDNF, and p-GluR1 levels were restored with chronic minocycline treatment [128, 165–168]. Peroxisome proliferator-activated receptor γ (PPARγ) is a nuclear receptor that regulates inflammation and microglial polarization and is a potential treatment target in MDD. Pioglitazone, a PPARγ agonist, significantly ameliorated depressive-like behaviors in CMS mice by regulating the expression of pro-inflammatory markers (IL-1β, IL-6, TNF-α, iNOS, and CCL2) together with anti-inflammatory markers (Ym1, Arg1, IL-4, IL-10, and TGF-β) in the HIP [169]. Other drugs that play a potential antidepressant role in modulating microglial phenotype are shown in Table 1.

### Plant-derived natural compounds and formulations with antidepressant properties

Recently, the regulatory properties of some plant-derived natural compounds and formulations have shown efficacy in treating depression. In this review, the antidepressive effect of natural products such as ginsenosides (Rg1, Rg3, and Rb1), resveratrol, salvianolic acid B, and magnolol on microglia-mediated neuroinflammation are summarized in Table 2. The chemical structures of several natural compounds that may act as potential inhibitors of pro-inflammatory microglial activation in MDD are illustrated in Fig. 5.

### Flavonoids

Flavones are subgroups of flavonoids composed of a backbone of 2-phenylchromen-4-ketone, and their biological activities have been reported in vitro and in vivo [184]. Flavonoids achieve their antidepressive effects through modulating microglial activation and NLRP3 inflammasomes. Baicalin, a flavonoid found in the root of *Scutellaria*, could ameliorate depressive behaviors in CMS model [185, 186]. Oral administration of baicalin (20, 40 mg/kg/day) for 3 weeks significantly improved sucrose intake, locomotor activities, and behavioral despair in CMS rats. The mechanism of baicalin against CMS-induced depression was partially achieved by increasing levels of DCX, neuron-specific enolase (NSE), and BDNF, reducing oxidative stress, and modulating the GSK3/NF-κB/NLRP3 signal cascade in the HIP [185]. In addition, baicalin supplementation (30, 60 mg/kg/day, p.o.) for 3 weeks also inhibited TLR4 activation and pro-inflammatory cytokine secretion in CMS mice. Furthermore, it was confirmed that the inhibition might be realized through PI3K/AKT/FoxO1 pathway in LPS-stimulated BV2 cells [186]. Quercetin is a flavanol present in apples, onions, and berries [37]. Treatment with quercetin (40, 80 mg/kg/d, p.o.) for 2 weeks significantly reversed OBX-induced increase in immobility time in the FST, possibly by inhibiting TNF-α, IL-6, and caspase-3 in the HIP and cortex. Co-treatment with minocycline (25 mg/kg, p.o.) significantly potentiated its protective effects when compared to treatment with quercetin alone [187]. Treatment with quercetin (40 mg/kg/day, p.o.) for 2 weeks alleviated depressive-like behavior in the SPT and FST of LPS-challenged rats via regulation of BDNF-related imbalance of Copine 6 and TREM1/2 in the HIP and PFC [188].

### Terpenoids

Terpenoids, especially triterpenoids, monoterpenes and sesquiterpenes, are the most abundant natural compounds found in several vegetables and fruits. Recently, some terpenoids have been reported to have preventive efficacy against neurological diseases by modulating the activation of microglia, especially on depression. Saponins, which are triterpene glycosides, are abundant in some plants, and their general biological activities have been summarized [189]. Saikosaponin-D is a triterpenoid saponin derived from *Radix bupleuri* and exhibits anti-inflammatory, anti-oxidative, and other pharmacological activities [190]. Previous studies have suggested that treatment with saikosaponin-D (0.75, 1.50 mg/kg/day, p.o.) for 3 weeks remarkably ameliorated the CMS-induced depressive-like behaviors in rats, mainly by improving HPA axis functions and hippocampal neurogenesis [191]. Moreover, additional research confirmed that saikosaponin-D administration ameliorated LPS-induced depressive-like behaviors, as shown by increased sucrose consumption in the SPT and decreased immobility time in the TST and FST. These performances appear to be mediated by inhibition of microglial activation and regulation of the high mobility group box 1 (HMGB1)/TLR4/NF-κB signaling pathway [190]. Gypenosides, the major ingredients of *Gynostemma pentaphyllum*, exert a neuroprotective function in the CNS. Treatment with gypenosides (50, 100 mg/kg/day, p.o.) for 4 weeks significantly relieved depressive-like behaviors of CMS mice in the TST and FST, which was mediated, in part, by inhibiting microglial activation and NF-κB signaling and increasing BDNF levels in the HIP [192, 193].

Ginsenosides are one of *ginseng*’s most biologically active ingredients and have a triterpenoid glycoside structure [194]. Direct and indirect evidence support that some ginsenosides (such as ginsenoside Rg3, Rb1, and Rg1) induce the anti-inflammatory actions of macrophages and microglia [195]. Ginsenoside Rb1 is a typical 20(*S*)-protopanaxadiol-type saponin and exerts significant antidepressive effects in chronic stress models [73, 196, 197]. It is known that Rb1 regulates microglial activation, protects neurons from inflammatory and oxidative damage, and promotes neurogenesis [198, 199]. Recently, treatment with Rb1 (20 mg/kg/d, *p.o.*) in CMS-treated mice for consecutive 4 weeks alleviated depressive-like behaviors in the SPT, TST, and FST mainly through PPARγ-mediated transitions in microglial phenotype (TNF-α, IL-1β, TGF-β, and Arg-1) in the HIP and cortex [73]. Rb1 attenuated a decrease in BDNF and the ratio of p-AKT/AKT expression, and increased IL-1β, TNF-α, and Iba-1 levels in the HIP in a CRS model. In line with in vivo reports, Rb1 lowered the protein expressions of IL-1β and TNF-α in BV-2 cells [197]. In addition to Rb1, ginsenoside Rg1 and Rg3 exhibited potential anti-inflammatory effects, which protected against microglial activation in stress-injured rodents [200, 201]. A previous study reported that chronic pretreatment with ginsenoside Rg1 (40 mg/kg/d, i.p.) for 5 weeks significantly suppressed inflammatory responses by alleviating microglial and astrocyte activation through decreased overexpression of IL-1β, IFN-γ, and TNF-α. These effects were accompanied by attenuation of dendritic spine and synaptic defects, and upregulation of synaptic-related proteins in the ventral medial prefrontal cortex (vmPFC). Ginsenoside Rg1 inhibited neuronal apoptosis by increasing Bcl-2 and decreasing cleaved caspase-3 and caspase-9 expressions after CMS exposure. Furthermore, ginsenoside Rg1 increased the nuclear factor erythroid 2-related factor (Nrf2) and inhibited p38 mitogen-activated protein kinase (p-p38 MAPK) and p65-NF-κB activation in the vmPFC [200]. Moreover, ginsenoside Rg3 at oral doses of 20 and 30 mg/kg attenuated upregulation of TNF-α, IL-1β, and IL-6 mRNA expression in brains of C57BL/6 mice after systemic LPS injection. Morphological activation of microglia, and Iba-1, cyclooxygenase-2 (COX-2), iNOS protein expression was reduced with Rg3 treatment [201]. Our previous studies also found that ginsenosides Rd and Re (10, 20, or 40 mg/kg, p.o.) have potentially neuroprotective and anti-inflammatory properties, as manifested by significantly reducing the expression of hippocampal pro-inflammatory factors and NLRP3 inflammasome related protein, as well as enhancing endogenous antioxidant factor Nrf2, and mediating PI3K-AKT and BDNF signaling pathways in mice exposed to CRS [129, 202]. Taken together, the anti-inflammatory effects of ginsenosides have been confirmed, and the negative regulation of pro-inflammatory cytokines and enzymes has been found to underlie the anti-inflammatory properties of ginsenosides in pro-inflammatory microglia in depression. However, additional studies are needed to investigate whether other ginsenosides or their metabolites can relieve depressive symptoms through modulating microglial phenotype and the molecular mechanisms involved in neuroinflammation.

### Iridoid glycosides

Catalpol, an iridoid glucoside, is primarily isolated from *Radix rehmannia* and is commonly used as a traditional Chinese medicine [203]. Previous studies have been reported that catalpol exhibits a wide range of pharmacological effects, including exhibiting anti-diabetic, anti-tumor, anti-inflammatory, and antioxidant activities. Growing evidence indicates that catalpol has a robust antidepressant effect that acts through its anti-inflammatory and anti-oxidative properties in vitro and in vivo [203, 204]. Catalpol (5, 10, or 20 mg/kg/day, i.g.) administration for 5 weeks ameliorated CMS-induced depressive-like behavior in the SPT, and its underlying mechanisms might be at least partially ascribed to reducing HPA axis dysfunction, upregulating BDNF and its specific binding receptor tyrosine kinase B (TrkB), downregulating COX-2 expression, thus reducing prostaglandin E2 level in the brain [204]. It was also confirmed that catalpol at 20 mg/kg decreased the expression of NLRP3 inflammasome-associated proteins and inhibited pro-inflammatory microglial polarization in the HIP (IL-1β, TNF-α, and iNOS) [205]. Geniposide, a type of iridoid glycoside extracted from the ripe fruit of *Gardenia jasminoides* Ellis, exhibits numerous bioactivities, including anti-diabetic, anti-oxidative, and anti-inflammatory actions [206]. It has been demonstrated that administration of geniposide (10, 40 mg/kg/day, p.o.) for 1 or 3 weeks ameliorated LPS- and CMS-induced depressive-like behavior in the SPT, TST, and FST by regulating the Bruton’s tyrosine kinase (BTK)/TLR4/NF-κB, BDNF/TrkB, and BTK/JAK2/STAT1 signaling pathways [207, 208]. Also, the beneficial effect of geniposide on anxiety- and depressive-like behaviors in mice may possibly act through induction of microglial polarization towards anti-inflammatory phenotype and inhibition of IL-6 and TNF-α release [208].

### Phenylpropanoids

Arctigenin and its glycoside, arctiin, are the major active ingredients of the dried ripe fruit of *Arctiumlappa* L. Arctiin can be metabolized into arctigenin by human intestinal microflora after oral consumption [209]. Arctiin has antidepressive effects that appear to act through inhibition of the NF-κB mediated HMGB1/TLR4 and TNF-α/TNF receptor 1 (TNFR1) pathways, which consequently attenuated microglial activation and neuroinflammation [210]. Similarly, arctigenin showed antidepressive effects by inhibiting microglial activation and neuroinflammation via HMGB1/TLR4/NF-κB and TNF-α/TNFR1/NF-κB signaling pathways [171]. Salvianolic acid B (SalB) is one of the phenolic acid compounds derived from *Salvia miltiorrhiza*, which has various pharmacological effects, including anti-inflammatory, antioxidant, and anti-apoptotic [211, 212]. CMS mice treated with SalB (20 mg/kg/day, i.p.) for 3 weeks significantly reversed decreased sucrose preference index in the SPT and increased immobility time in the FST and TST. In addition, the decreased expression of IL-1β and TNF-α and increased expression of IL-10 and TGF-β were accompanied by increased apoptosis (cleaved-caspase 3^+^) and microglial activation (Iba-1^+^) in the HIP and cortex that were reversed after SalB treatment [213]. Curcumin is a representative natural phenolic compound of turmeric and a potential candidate for regulation of brain function due to its antioxidant and anti-inflammatory properties. In a CRS model, systemic administration of curcumin (10, 20, or 30 mg/kg, i.p.) daily for 3 weeks significantly attenuated oxidative stress and lipid peroxidation, prevented apoptosis, and increased antioxidant defense activity [214]. Curcumin (10 mg/kg, i.p.) also was beneficial for maintaining improved memory and mood functions after 30 days of daily therapy in a Gulf War Illness (GWI) with stress-treatment model. At the molecular level, enhanced numbers of DCX^+^ cells, decreased numbers of Iba-1/ED-1^+^ cells, and elevated antioxidant genes with normalized mitochondrial respiration might be the basis of the regulatory mechanism that is mediated by curcumin treatment [215]. Magnolol, a hydroxylated biphenyl compound extracted from Magnolia tree, has endothelial cell protective, antioxidant, and anti-inflammatory functions [216]. It is noteworthy that magnolol exhibits antidepressant effects, as evident by increased sucrose consumption and decreased the immobility time in the SPT, TST, and FST. In an OBX model, magnolol (50, 100 mg/kg/day, p.o.) treatment for 2 weeks produced antidepressant-like effects by enhancing neurogenesis (as indicated by BrdU positive cells) in the HIP [217]. In a CMS model, treatment with magnolol (20, 40 mg/kg/day, p.o.) for 4 weeks increased BDNF expression and normalized serotonergic system [218]. In agreement with previous studies, the protective effect of magnolol (50, 100 mg/kg/day, p.o.) given for 3 weeks to CMS-treated female mice also appeared to be associated with inhibition of pro-inflammatory microglia (TNF-α, IL-1β, and IL-6) and activation of anti-inflammatory microglia (IL-4, IL-10, Arg1, Ym1, Fizz1, and Klf4) via Nrf2/hemeoxygenase (HO-1)/NLRP3 signaling pathway [219].

### Others

Resveratrol is one of the most well-known dietary stilbenoids. Resveratrol confers health benefits due to its antioxidant, anti-inflammatory, anti-aging, and immune-regulatory activities [220]. Resveratrol is found in the skin of red grapes, peanuts, and other medicinal plants. Recent studies have found that resveratrol ameliorated depressive-like behaviors in several animal models [221–223]. In a LPS model, mice were treated for 2 weeks with resveratrol (20 mg/kg/day, i.p.), which abrogated the increased immobility in the FST and TST. Immunohistochemical staining revealed that resveratrol reversed increased microglial activation (Iba-1^+^) and inhibition of neurogenesis (BrdU/DCX^+^) in the DG [224]. Moreover, 3 doses of resveratrol (20 mg/kg/day, i.p.) promoted the activation of sirtuin type 1 and blocked the decline of hippocampal neurogenesis triggered by ethanol exposure during early postnatal life [225]. The carotenoids crocin was able to regulate microglial activation associated with neurological disorders. Crocin decreased the expression of LPS-induced NO, TNF-α, and ROS production in BV-2 cells and improved locomotor activity, sucrose intake, and reduced immobility time in LPS-treated Kunming mice [226]. The health benefits associated with crocin might be due to its potent ability to regulate the NLRP3 inflammasome and NF-κB, as well as anti-inflammatory phenotypic conversion [226]. Moreover, emerging data have shown that plant-derived compounds can exert neuroprotective effects that prevent neurological disorders in depression. For instance, ganoderic acid A [227], asperosaponin VI [228], 20(*S*)-protopanaxadiol [229], scutellarin [230], astragalin [231], hesperidin [232], 5-*O*-methylvisammioside [233], ginkgolide B [234], leonurine [235], ferulic acid [236], and (+)-sesamin [69] were found to inactivate microglia in stress-induced depression models. Recent information concerning the antidepressant activity of phytochemicals through regulation of microglial polarization is illustrated in Table 2. In addition, several herbal extracts and traditional formulations reported to improve depression-like behavior by regulating microglia are shown in Table 2 [237–245].

## Conclusions

**Fig. 4:**
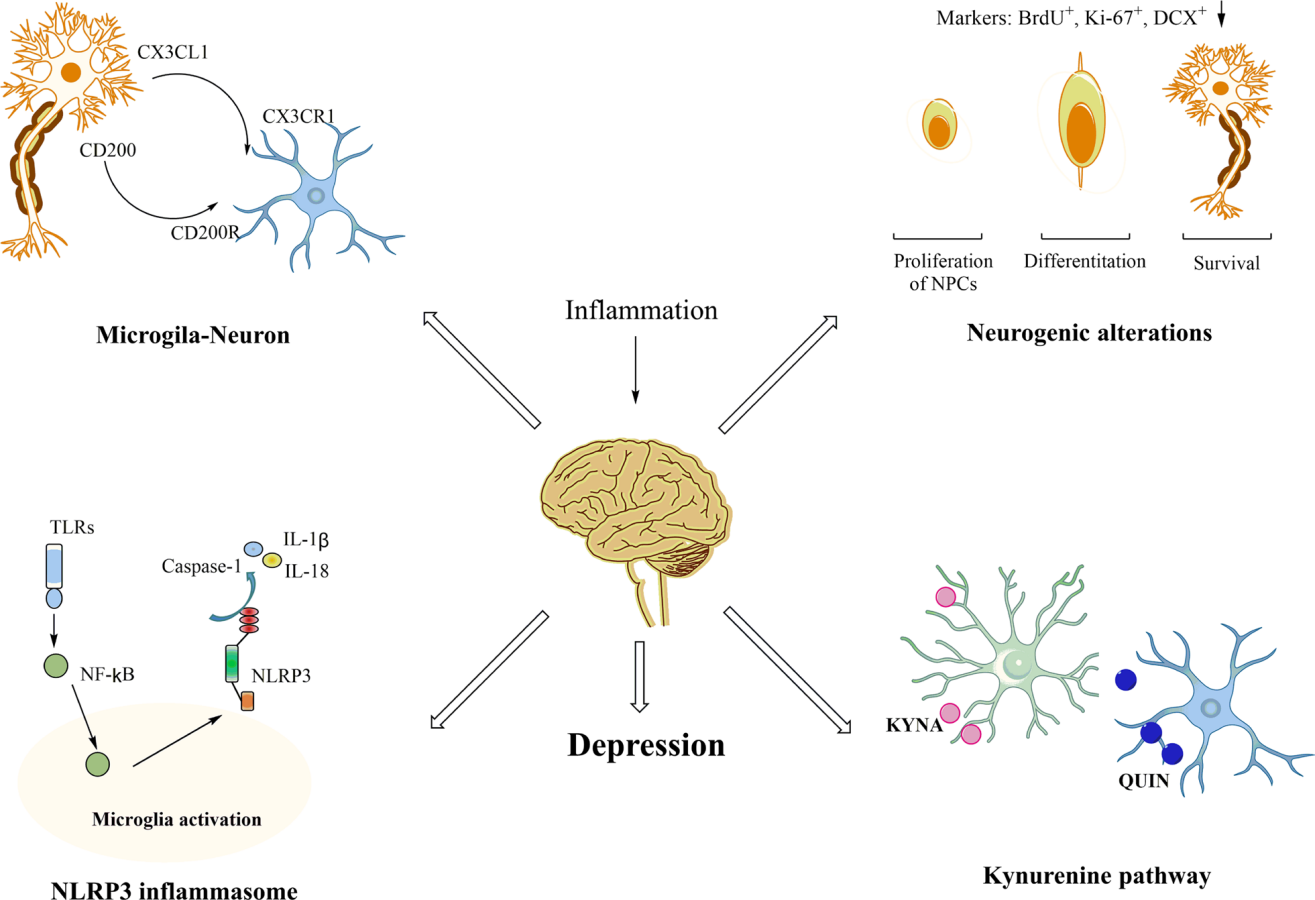
Schematic diagram of microglial activation involved in the pathogenesis of depression. Dysfunction of neuron–microglia interactions, particularly the CX3CL1-CX3CR1 and CD200-CD200R interactions, lead to behavioral and biochemical alterations. In addition, the most relevant markers of the NLRP3 inflammasome and neurogenesis used in the reviewed studies are indicated. We also included the generation of neuroactive kynurenine metabolites in depression disorders

This review has summarized the current knowledge regarding microglial activation in depression as seen in clinical and preclinical studies. Microglia are the primary resident immune cells that are currently considered as a critical link between neurological and immunological activity in the CNS [247]. These cells modulate neuronal function not only during an inflammatory response, but also during developmental synaptic pruning and plasticity in healthy brains [27]. The heterogeneous states of activated microglia exist on a continuum ranging from neuroprotection to neurotoxic/pathogenic activity (Figs. 1 and 2) [248]. In this review, we summarized microglial activation in depressed patients and animal models (Additional file 1: Table S1), as well as the possible mechanisms associated with the pathogenesis of MDD (Fig. 4). Moreover, we described the therapeutic role of traditional antidepressants and phytochemicals in stress-induced depression models (Tables 1 and 2).

**Fig. 5:**
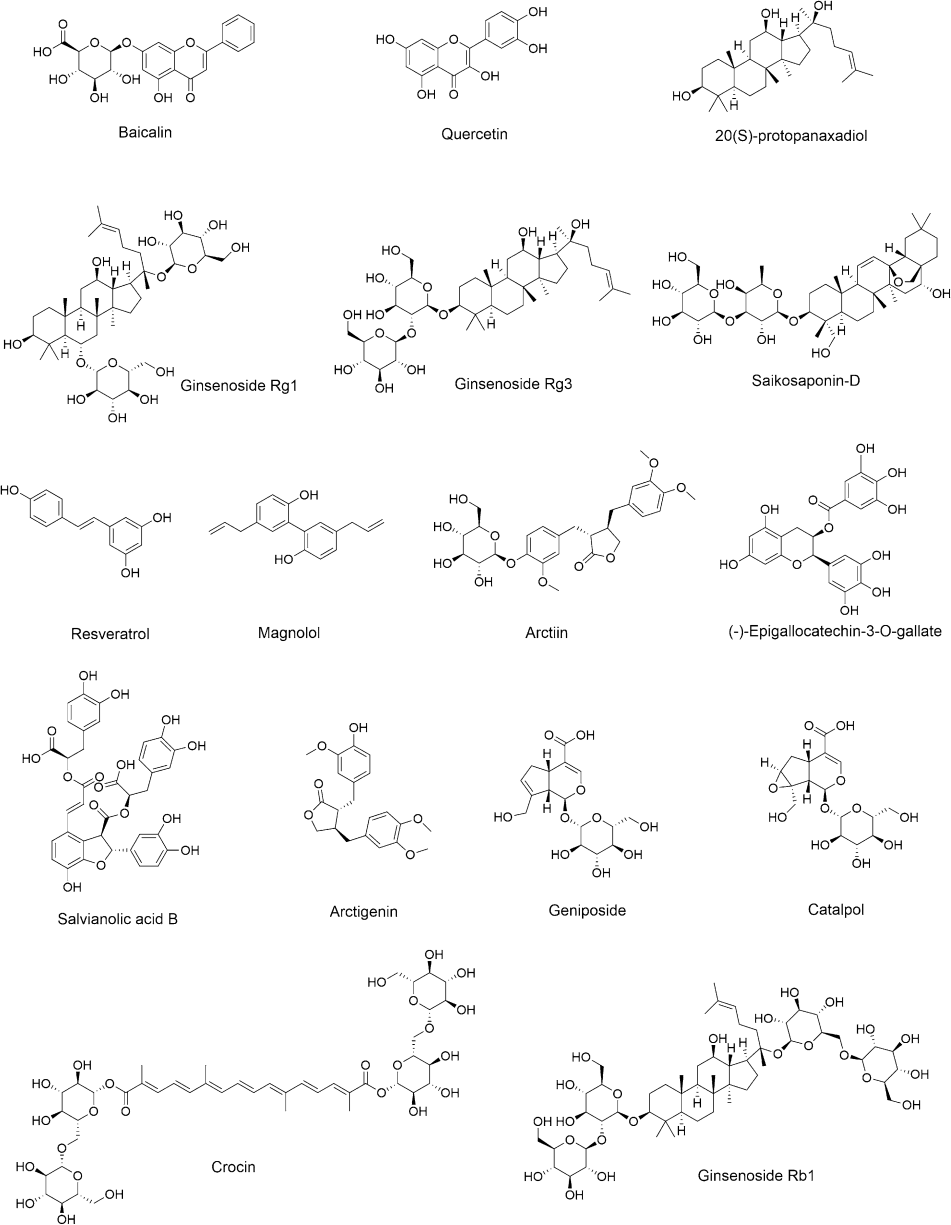
Chemical structures of several plant-derived natural compounds that act as potential inhibitors of M1-type microglia activation in animal models of depression

Recently, natural compounds have been considered to be potential agents for the prevention or treatment of neuropsychiatric diseases due, in part, to their antioxidant and anti-inflammatory activities [249]. Numerous studies have shown that natural compounds and formulations are beneficial in the treatment of depression through their ability to regulate microglial functions, as listed in Table 2. The chemical structures of these bioactive compounds are highlighted in Fig. 5. Most compounds have been shown to modulate inflammatory response, oxidative stress, and ameliorate symptoms of depression through inhibiting microglial activation. However, additional research is needed to determine whether these compounds promote the transformation of microglia with a pro-inflammatory phenotype to an anti-inflammatory phenotype, or whether they depend on regulation of downstream signaling pathways that are activated by microglia. In a pathological state, changes in microglial phenotypes depend on the disease stage and severity. Therefore, developing protocols that control the stage-specific conversion of pro-inflammatory/anti-inflammatory actions in appropriate time frames might provide better therapeutic outcomes. Thus, changing the activity of microglia to a stable state through pharmacological or non-pharmacological methods will be the focus of future research, which also may provide new ideas for targeted treatment of depression. Moreover, it has been shown that some polyphenolic compounds are not completely absorbed via the gastrointestinal tract but still possess anti-inflammatory actions due to metabolites that are produced by intestinal microflora [250]. However, information concerning the role of these activated metabolites through microglia regulation is still minimal. Therefore, the discovery of potential inhibitors against microglial activation from metabolites may also be another promising strategy for the prevention of depression in the future.

In addition, data primarily obtained from studies that used specific microglial manipulation methods help improve our understanding of the mechanisms underlying neurogenesis, inflammation, and neurotransmitter metabolism dysfunction in depression. Genetic and pharmacological methods that directly or indirectly modify the activation status of microglia have been used and achieved some success, such as the *Cx3cr1*^−/−^ or *Cd200*-deficient mouse models and the minocycline or PLX4497 treatment models [75, 251]. Moreover, it should be noted that the dichotomy of microglial activation states (M1/M2) is an oversimplified conceptual framework. This dichotomy is not generally accepted as it may not accurately reflect the heterogeneous microglial profiles that can be observed in complicated homeostatic or disease conditions [252–254]. The spatial and temporal distribution of microglial subsets can be better assessed using newly developed unbiased and high-throughput methods. Novel single-cell techniques enable scientists to overcome such limitations and reveal the surprising context-dependent heterogeneity of microglia [247, 255]. Specific subsets and key targets of microglia have been revealed in the development of AD and regulators such as TREM2 that control disease severity has been identified [42]. Currently, advanced technologies and tools are being used to comprehensively decipher the role of microglial heterogeneity in the pathology of depression, particularly in the modulation, inhibition, and stimulation of contextually relevant microglia functions. These technologies also are being used to target microglia to explore the treatment of neuropsychiatric diseases, which remains to be explored further.

Taken together, the data analyzed in this review suggest that microglial changes mainly affect the regulation of inflammatory response, neurogenesis, and tryptophan metabolism with respect to the development of depression. However, other factors should not be ignored. The discovery of natural compounds that exert antidepressant effects by inhibiting microglial activation may contribute to the effectiveness of preventing and treating depression. Additional information regarding the neuroprotective properties of these compounds that act through the regulation of microglial phenotypes remains to be explored in the future. High-performance omics technologies are expected to provide more effective molecular targets and identify additional specific signaling pathways in drug screening and disease diagnosis.

## Supplementary Information

**Additional file 1: Table S1.** The animal models of depression involving microglial changes.

## Data Availability

Not applicable.

## Abbreviations

MDD: Major depressive disorder
COVID-19: Coronavirus disease 2019
HPA: Hypothalamus–pituitary–adrenal
CNS: Central nervous system
PFC: Prefrontal cortex
HIP: Hippocampus
vHIP: Ventral hippocampus
BLA: Basolateral
SN: Substantia nigra
NPC: Neural progenitor cell
OPC: Oligodendrocyte progenitor cell
CD86, CD16/32: Differentiation marker 86 and 16/32
iNOS: Inducible nitric oxide synthase
Arg1: Arginase-1
TGF-β1: Transforming growth factor-β1
BDNF: Brain-derived neurotrophic factor
DAM: Disease-associated microglia
PAMPs: Pathogen-associated molecular patterns
DAMPs: Danger-associated molecular patterns
TLRs: Toll-like receptors
LPS: Lipopolysaccharide
IFN-γ: Interferon-γ
IL-1β: Interleukin-1β
TNF-α: Tumor necrosis factor-α
ROS: Reactive oxygen species
ACC: Anterior cingulate cortex
QUIN: Quinolinic acid
PET: Positron emission tomography
Iba-1: Ionized calcium-binding adapter molecule 1
CMS: Chronic mild stress
CSDS: Chronic social defeat stress
CRS: Chronic restraint stress
OBX: Olfactory bulbectomy
LH: Learned helplessness
NLRP3: Nod-like receptor pyrin containing 3
NF-κB: Nuclear factor-kappa B
SPT: Sucrose preference test
FST: Forced swimming test
TST: Tail suspension test
LD: Light–dark
SIT: Social interaction test
NORT: Novel object recognition test
AD: Alzheimer’s disease
SGZ/GCL: Subgranular zone/granule cell layer
CWIRS: Chronic water-immersion restraint stress
NMDAR: N-Methyl-d-aspartate receptor
SYP: Synaptophysin
PSD95: Postsynaptic densitin-95
SSRIs: Selective serotonin reuptake inhibitors
TCAs: Tricyclic antidepressants
TRP: Tryptophan
KP: Kynurenine pathway
KYNA: Kynurenic acid
3-HK: 3-Hydroxykynurenine
KYN: Kynurenine
NSFT: NOvelty suppressed feeding test
ST: Splash test
IDO1: Enzyme indoleamine 2, 3-dioxygenase 1
PSD: Poststroke depression
BDNF: Brain-derived neurotrophic factor
HAAO: 3-Hydroxyanthranilate 3, 4-dioxygenase
NAc: Nucleus accumbens
LTP: Long-term potentiation
PPARγ: Peroxisome proliferator-activated receptor γ
NSE: Neuron-specific enolase
vmPFC: Ventral medial prefrontal cortex
COX-2: Cyclooxygenase-2
TrkB: Tyrosine kinase B
BTK: Bruton’s tyrosine kinase
HMGB1: High mobility group box 1
TNFR1: TNF receptor 1
SalB: Salvianolic acid B
GWI: Gulf war illness
