# Supplementary material for: Microglia in depression: an overview of microglia in the pathogenesis and treatment of depression
Source: J Neuroinflammation. 2022 Jun 6;19:132. doi: 10.1186/s12974-022-02492-0 (PMC9168645; doi:10.1186/s12974-022-02492-0)
Supplement: Supplementary file 1 — Additional file 1: Table S1. The animal models of depression involving microglial changes. [file 12974_2022_2492_MOESM1_ESM.docx]

**Table S1** The animal models of depression involving microglial changes.

| Category | | | | Animal models | | | | Time points and strain | | | | | Behavioral test | Mode of action and potential signaling target |
| --- | --- | --- | --- | --- | --- | --- | --- | --- | --- | --- | --- | --- | --- | --- |
| Acute stress models | | | | — | | | | Male Gunn rats and Wistar rats (7 weeks) | | | | | FST, TST | Iba-1^＋^↑ in DG, CA1, CA3 of HIP[1] |
|  | | | | Focal excitotoxic lower motor neuroninjury (Glutamatemicroinjection) ＋ acute restraint stress | | | | 60 min, adult female SD rats (225–250 g) | | | | | Paw preference test | OX42/BrdU^＋^↑ in brain[2] |
|  | | | | Acute restraint stress | | | | 6 h, young adult male Wistar Hannover rats (12 weeks, 225–250 g) | | | | | — | mRNA: 4-HNE, KEAP ↑;  Proteins: IL-4 ↑, IL-10 ↓ in PFC[3] |
|  | | | | Acute alcohol ＋restraint/water immersion stress | | | | 2 h, male Wistar rats (10 weeks) | | | | | — | Plasma CORT, endotoxin ↑;  CD11b^+^, c‐Fos^+^↑ in prelimbic cortex (Plx)[4] |
|  | | | | Acute restraint stress | | | | 1–4 h, male Fischer rats (250–280 g) | | | | | — | Plasma CORT ↑; Iba-1^+^, OX42/DBH^+^ ↑ in HIP, thalamus, hypothalamus; Proteins: β1-AR, β2-AR ↑ in hypothalamus[5] |
| Sub-chronic stress models | LH | | | | | | | 2 d, male SD rats (200–230 g) | Two-way conditioned avoidance test | | | | | OX42^+^↑ in [granule cell](https://www.sciencedirect.com/topics/neuroscience/granule-cell)layer, hilus, CA3 of HIP[6] |
|  |  | | | | | | | 2 d, male IL-4^-/-^ mice (BALB/cJ background) | SPT, FST, LD | | | | | MHC II^+^, CD200R^+^↑ in isolated microglial[7] |
|  |  | | | | | | | 2 d, male integrin β4^-/-^mice | OFT, EPM, SPT, FST, LHT, amphetamine-inducedhyperactivity | | | | | IL-1β^+^, IL-6^+^, TNF-α^+^ ↑ in HIP and PFC;  ELISA: IL-4, IL-13 ↑ in serum; Proteins: Iba-1,TNFRα ↑, cellapoptosis ↑, synaptophysin ↓ in HIP and PFC[8] |
|  |  | | | | | | | 2d, male C57BL/6mice (8–12weeks) | — | | | | | Iba-1^+^ ↑ in DG of HIP[9] |
| Surgical-induced models | | | OBX | | | | | Male SD rats  (175–200g) | | | OFT | | | mRNA: CD11b, IL-1β ↑ in amygdala[10] |
|  | | |  | | | | | Male Wistar rats (250–300g) | | | OFT, FST | | | Serum CORT ↑;  ELISA: TNF-ɑ，IL-6 ↑;  Proteins: Caspase-3 ↑ in cortex and HIP[11] |
|  | | |  | | | | | Male SD rats, (175–200g) | | | OFT | | | mRNA: CD11b, IL-1β ↑, MRC2, IL-10 ↓ in PFC[12] |
|  | | |  | | | | | ddY mice (28–32 g) | | | Emotional behavior, FST, TST | | | BrdU/ NeuN^+^↓, DCX/NeuN^+^↓ in DG;  Iba-1^+^, GFAP^+^↑ in HIP;  Proteins: [tyrosine hydroxylase](https://www.sciencedirect.com/topics/neuroscience/tyrosine-hydroxylase) (TH), p-TH, p-PKA, p-DARPP-32, p-ERK1/2, p-CREB, BDNF, DCX, NeuN, Bcl-2 ↓, Iba-1, GFAP, p-IkB-ɑ, p-p65 NF-κB, TNF-ɑ, IL-6, Bax, cleaved-Caspase-3 ↑ in HIP[13] |
|  | | |  | | | | | Fat-1 positive C57BL/6 mice (8 weeks) | | | OPT, SPT | | | mRNA: CD11b, TNF-ɑ, IL-6 ↑ in HIP[14] |
| Chronic stress models | CRS | | | | | | 20 h/week for 6 weeks, C57BL/6 adult male mice (8 weeks) | | | | | — | | TH^+^, Iba-1^+^, GFAP^+^↓;  Proteins: protein carbonylation, TH, CX3CR1, Iba-1, GFAP, S100b, soluble ɑ-synuclein monomer ↓, insoluble ɑ-synuclein monomer ↑ in substantia nigra [15] |
|  |  | | | | | | 2*30 min/d, 2 weeks, male SD rats | | | | | Struggling behavior during stress, SPT | | The density Iba-1^＋^↑ (nine regions);  The number of Iba-1^＋^↑ (eight regions)[16] |
|  |  | | | | | | 5 h/d for 3 weeks, male Mongolian gerbils (24 weeks) | | | | | — | | Iba-1^＋^↑ in HIP[17] |
|  | Cerebral ischemia＋CRS | | | | | | 5 h/d for 3 weeks, male progeny of Mongolian gerbils (24 weeks, 65–75g) | | | | | OFT | | Cu, Zn-superoxide dismutase^＋^↑ in stratum pyramidale[18] |
|  |  | | | | | | 6 h/d for3 weeks, adult male SD rats (350–450 g) | | | | | — | | Iba-1^＋^↑ in Plx and infralimbic cortex (IL)[19] |
|  |  | | | | | | 30 min/d, 2 weeks, male SD rats (250–275 g) | | | | | — | | Iba-1^＋^↑ in Plx and IL[20] |
|  |  | | | | | | 2h/d, 2 weeks, female C57B1/6J (8 weeks) | | | | | — | | Iba-1/Cox-2^＋^↑;  Proteins: Bax, Cox-2 ↑ in cortex and HIP[21] |
|  | CSDS, CRS, TS | | | | | | CSDS for 30 min, CRS for 2 h, TS for40 min, male C57BL/6Jmice (14 weeks) | | | | | SPT, FST, LD | | Iba-1^＋^↑ in PFC; mRNA: TNF-ɑ↑ in PFC;  mRNA: IL-1β ↑ in HIP;  Serum CORT ↑;  5-HT_2A_ ↑ in PFC[22] |
|  |  | | | | | | 2 h/d for 10 d, Wild-type C57BL/6 or Panx1^-/-^ male mice | | | | | OFT, LD | | Panx1/Iba-1^＋^↑, NMDA, P2X7R, ATP, glutamate release ↑ in HIP slices[23] |
|  | Focal excitotoxic lower motor neuron injury＋chronic variable stress | | | | | | 2 h/d for 2 weeks, adult female SD rats (225–250g) | | | | | PPT | | Neuron loss[2] |
|  |  | | | | | | 6 h/d for 3 weeks, young adult male Wistar Hannover rats (12 weeks, 225–250 g) | | | | | — | | mRNA: IL-4 , IL-10 ↓;  Proteins: 4-HNE ↑, Nrf2, PI3K, HO-1, Arg-1 ↓ in PFC[3] |
|  |  | | | | | | 20 h/d for 3 weeks, adult male ICR mice | | | | | OFT, TST, FST | | Lactobacillus, Firmicutes ↑, Bacteroidetes, Proteobacteria ↓ in fece;  Proteins: Iba-1, IL-1β, TNF-α, p-NF-κB p65 ↑, BDNF,p-AKT/AKT ↓ in HIP;  ELISA: IL-1β, TNF-α ↑ in serum[24] |
|  |  | | | | | | 6 h/d for 3 weeks, male Wistar rats (8 weeks, 200–210 g) | | | | | OFT,TST,FST | | Serum CORT;  Proteins: Iba-1, ROS, NF-κB, NLRP3, cleaved caspase-1, IL-1β, IL-18 ↑ in HIP[25] |
|  | Chronic water immersion restraint stress (CWIRS) model | | | | | | 2 h, restraint＋ water bath (21 °C), Kunming male mice (8 weeks, 28–30 g) | | | | | SPT, TST, FST | | BrdU/DCX^+^↓, CD68^+^↑ in DG;  5-HT ↓, 5-HIAA ↑; Plasma CORT↑;  Proteins: iNOS, TNF-α, IL-1β, MEK, ERK, NF-κB ↑ in HIP [26] |
|  | Middle cerebral artery occlusion model (MCAO)＋stress | | | | | | 2 h/d for 2 weeks, male ICR mice (28–30 g) | | | | | TST, FST | | IL-18, Iba-1^+^↑ in brain at day 7;  Proteins: IL-18 ↑ in cortex and amygdala[27] |
|  | GWI＋stress | | | | | | GWIrelated chemicals (GWIR-Cs)＋ stress (5min, 28 d), male SD rats (9 weeks) | | | | | OLT, NORT, NSFT | | BrdU^+^↓, DCX^+^↓, net neurogenesis↓ in subgranular zone-granule cell layer;  Iba-1/ED-1^+^↑ in HIP[28] |
|  | CWIRS | | | | | | 2 h/d for 4 weeks, adult male SD rats (260–270 g) | | | | | OFT, SPT, TST, FST | | Iba-1^+^, Iba-1/iNOS ^+^↑, Iba-1/Arg1^+^↓ in CA1;  Proteins: Iba-1, IL-1β, IL-6, iNOS ↑, IL-4, IL-10, Arg1 ↓ in HIP[29] |
|  |  | | | | | | 30 min/d for 10 d; CX3CR1-GFP, C57BL/6J male mice (8–12weeks) | | | | | LD, three-compartment sociability test, SPT, social dominance test, Morris water maze test (MWM) | | CD206^+^↑ in HIP and PFC;  VGLUT2/CD206^+^↑ in HIP;  Microglial process area ↓, circularity and solidity ↑ in anterior part of the basal lateral AMYG (BLA)[30] |
|  |  | | | | | | 4 h/d for 3 weeks; adult male ICR mice | | | | | OFT, TST, FST | | IL-1β^+^, TNF-α^+^, lectin^+^, BDNF^+^↓ in CA3;  Proteins: BDNF, p-AKT/AKT ↓, IL-1β, TNF-ɑ, Iba-1, lectin ↑ in HIP[31] |
|  |  | | | | | | 6 h/d for 4 weeks, male Wistar rats (8 weeks, 250–270 g) | | | | | SPT, NSFT, OFT, FST | | Iba-1^+^↑ in PFC;  Proteins: p62 ↑, beclin1 ↓, LC3-II ↓, p-AKT/AKT ↑, p-mTOR/mTOR ↑, p-ULK1/ULK1 ↑, p-AMPK/AMPK ↓, Iba-1 ↑, NLRP3, ASC, caspase-1 ↑;  mRNA: NLRP3, IL-1β, IL-6, IL-18, TNF-α ↑ in PFC[32] |
| Chronic stress models | | | CSDS | | | 5 min/d for 2 weeks, adult male C57BL/6 mice (10–13 weeks); adult male Rag*^-2-^* mice | | | | LD, OFT, SI, Urine scent-marking (USM), TST | | | | Plasma CORT, TNF-ɑ, IL-1β, IL-2, IL-3, IL-6, IL-17, IFN-γ ↑;  Proteins: M1(IL-1β, IL-6) ↑, M2 (MRC1, muted ARG) ↓ in cells from each brain[33] |
|  | | |  | | | 5 min/d for 3 d or 14 d; CD-1, C57BL/6J, Cx3cr1wt/gpf, interbred Ccr2wt/rfp Cx3cr1wt/gpf mice | | | | SI, USM | | | | CD68hi^+^↑ in isolated microglia;  CX3CR1-GFP^+^ microglial ↑[34] |
|  | | |  | | | 10 min/d for 10 d, male C57BL/6J (7 weeks) and CD-1 mice | | | | Social avoidance test (SAT) | | | | NLRP3/Iba-1^+^↑, CX3CR1/Iba-1^+^↑, BrdU and DCX^+^↓ in DG of HIP;  IL-6^+^ microglia↑[35] |
|  | | |  | | | 20 d, male ICR mice | | | | TST, FST, FST | | | | Iba-1^+^↓;  Proteins: Iba-1, CD11b ↓ in DG[36] |
|  | | |  | | | 5 min/d for 2 weeks, male C57BL/6 and CD-1 mice | | | | SI, USM | | | | Infammation, phagocytosis, oxidativestress, extracellular matrix remodeling[37] |
|  | | |  | | | 5 min/d for 10d, WT and ZnT3 KO mice, on a mixed C57BL/6 × 129Sv background | | | | SI, EPM, NSFT, Y-maze, Conditioned fear | | | | Microglial density and morphology (–)[38] |
|  | | |  | | | 5 min/d for 10 d,male C57BL/6 mice (8–12 weeks, 20–25 g) and male CD-1 mice (16–20 weeks , 35–40 g) | | | | SRT, SPT, FST, TST | | | | Iba-1/iNOS^+^↑ in HIP;  Firmicutes ↓ in colon;  mRNA: FFAR2, FFAR3 ↓, TJP ↑ in HIP; IL-6, Proteins: IL-1β, TNF-ɑ ↑[39] |
|  | | | Mild early life stress ＋ CSDS | | | PND10-PND13 for 10 trials/day ＋ 4 months, 45 min/d, 3 weeks, 5 days per week | | | | — | | | | Iba-1^+^↑ in HIP hillus and the medial orbital PFC; PPARγ^+^↑ in HIP hillus[40] |
|  | | |  | | | 10 min/d for 10d, male C57BL/6J (7–8 weeks) and ICR mice (> 40 g) | | | | OFT, SAT, EPM, TST, FST | | | | TSPO/Iba-1^+^↑ in basolateral amygdale, lateral habenula, vHip[41] |
|  | | | Chronic stress ＋ ischemic brain injury | | | 5 min of physical interaction and followed by 25 min of threat for 8 d, male SD rats (250–275 g) | | | | — | | | | HMGB1/CD11b^+^↑ in CA1;  Serum HMGB1 ↑ at 7 days post-ischemia in HIP;  Proteins: NLRP3, ASC, Csapase-1, IL-1β, P2X7 IκB/IκB ↑ at 7 or 14 days post-ischemia in HIP[42] |
|  | | |  | | | 10 min/day for 10 d, male adult C57BL/6 mice (8 weeks, 20–25 g), CD1mice (14 weeks, 40–45 g) | | | | SI, OFT, FST , SPT | | | | —[43] |
|  | | |  | | | 24 h before the first social defeat (day 0) and 24 h after the 7 th episode of social defeat (30 min/d), male SD rats (275–300 g) and Male Long–Evans (LE) retired breeders (600–800 g) | | | | SI, FST, TST | | | | Iba-1^+^↑;  Protein: IL-1β ↑ in vHIP[44] |
|  | | |  | | | 5–10 min/d for 10 d, Male Cnr1^-/-^ and Cnr1^+/+^ (8–10 weeks), Male CD1 aggressor mice | | | | SAT, SPT | | | | Iba-1^+^↑ in DG; CD11b^+^, CD40^+^, MHCII^+^ on microglia ↑[45] |
| Chronic stress models | | | CMS | | | 45 days CMS, female SD rats (8–12 weeks, 280–300 g) | | | | | OFT, SPT, FST | | | ELISA: IL-1β, TNF-α, IL-6 ↑; total cholesterol, CORT ↑ in serum; Proteins: Bax ↑, bcl-2 ↓, GDNF, NGF ↓, NF-κB, P38 ↑, CD11b ↑, GFAP ↓, BDNF, TrkB ↓ in HIP[46] |
|  | | |  | | | 6 weeks CMS, male C57BL/6 mice (21–22 g) | | | | | SPT, TST, FST, OFT | | | Iba-1^+^↑ in DG; mRNA: IL-1β , IL-6, TNF-α, iNOS, CCL2 ↑, Ym1, Arg1, IL-4, IL-10, TGF-β ↓ in HIP[47] |
|  | | |  | | | 8 weeks CMS, male ICR mice(18–22 g) | | | | | SPT, TST, FST, OFT | | | Proteins: IL-1β, IL-6, TNF-α, TLR4 ↑, p-PTEN, p-PI3K, p-AKT, p-FoxO1 ↓ in HIP; Protein: p-FoxO1 ↓ in cytoplasmic fractions, Protein: FoxO1 ↑ innuclear extractions[48] |
|  | | |  | | | 8 weeks CMS, adult male C57BL/6J mice (18–22 g) | | | | | SPT, TST, FST, OFT | | | Iba-1^+^↑ in HIP and cortex; Brdu/DCX/Brdu^+^↓ in DG; mRNA: TNF-α, IL-1β ↑, TGF-β, Arg-1 ↓, PPARγ ↓ in HIP and cortex[49] |
|  | | |  | | | 7 weeks CMS, male C57BL/6 mice (18–22 g) | | | | | SPT, TST, FST, OFT | | | 7 weeks: Iba-1^+^ ↑ in HIP and cortex; mRNA: CD11b ↑ in HIP and cortex; IL-1β, TNF-ɑ, IFN- γ, IL-6 ↑; IL-10 ↑, IL-4, TGF-β, Ym-1 ↓ in HIP; BrdU/DCX^+^ ↓ in DG; 6 weeks: mRNA IL-1β, IL-6, iNOS ↑; Arg-1, IL-10 ↑, IL-4, TGF-β, Ym-1 ↓; 5 weeks: mRNA IL-1β, IL-6, IFN- γ, iNOS ↑; Arg-1, TGF-β, Ym-1 ↓[50] |
|  | | |  | | | 5 weeks CMS, C57BL/6 male mice (19–22 g) | | | | | FST, OFT, EMP | | | Iba-1^+^↑; ROS ↑ in HIP; mRNA: IL-1β, TNF-α, iNOS, IL-6; NLRP3, cleaved caspase-1, cleaved IL-1β ↑ in HIP[51] |
|  | | |  | | | 4 weeks CMS, male BALB/c mice (8 weeks) | | | | | SPT, TST, OFT | | | Proteins: Iba-1, IL-1β, TNF-ɑ, IL-6, iNOS, P2X7R ↑ in HIP and mPFC; ELISA: IL-1β, TNF-ɑ ↑ in serum[52] |
|  | | |  | | | 3 weeks CMS, BALB/c male mice (8 weeks, 22–24 g) | | | | | TST, FST, OFT | | | Iba-1^+^ ↑ in CA1, CA3, DG; ELISA: TNF-α, IL-1β ↑ in HIP; CORT ↑ in serum [53] |
|  | | |  | | | 3 weeks CMS, maleWistar rats (adolescent and adult) | | | | | SPT, OFT, EMP, MWM | | | Iba-1^+^↑, iNOS^+^↑; ELISA: IL-1β, IL-6 ↑; Proteins: iNOS, Jmjd3, H3K27me3 ↑ in PFC and HIP[54] |
|  | | | MCAO＋CMS | | | 3 weeks CMS, adult SD rats (250–300 g) | | | | | SPT, OFT | | | BDNF/CD11b^+^, TrkB/CD11b^+^↓ in amygdala[55] |
|  | | |  | | | 5 weeks CMS, male C57BL/6J mice (6–8 weeks, 25–30 g) | | | | | SPT, FST, TST, OFT | | | Iba-1^+^↑; Proteins: iNOS, IL‑1β, TNF‑α, IRAK1, TRAF6, p‑NF‑κB, p65-NF‑κB ↑ in HIP[56] |
|  | | | MCAO＋CMS | | | 3 weeks CMS, male SD rats (240–260 g) | | | | | SPT, OFT | | | ELISA/mRNA: TNF-α, IL-1β, IL-6 ↑, IL-10 ↓; mRNA: iNOS, CD86↑, Arg-1, CD206↓; Proteins: iNOS, CD86↑, Arg-1, CD206↓, p-ERK/ERK, p-CREB/CREB, BDNF, NGF ↓ in HIP[57] |
| Pharmacological rodent models | | LPS | | | LPS (0.83 mg/kg, *i.p.*) for 5 d, male C57BL/6Jmice (6–8 weeks, 25–30 g) | | | | | | | SPT, FST, TST, OFT | | Iba-1^+^ ↑;  Proteins: iNOS, IL‑1β,TNF‑α, IRAK1, TRAF6, p‑NF‑κB p65/NF‑κB p65 ↑ in HIP[56] |
|  | |  | | | LPS (0.5 mg/kg, *i.p.*), adult CX3CR1^-/-^ mice (3–6 m) | | | | | | | OFT, social exploratory behavior, resignation behavior | | IL-1β, CX3CL1 ↑ , KYN/TRP, 5-HIAA/5-HT, HVA/DA ↑ in plasma and cortex;  mRNA: IL-1β, TNF-ɑ, TLR2, CD14, CD11b, IDO, KMO ↑ from enriched microglia[58] |
|  | |  | | | LPS (0.33 mg/kg, *i.p.*), male adult BALB/c mice (3–4 m) | | | | | | | — | | mRNA: CD86, iNOS, IL-1β ↑, Ym-1 ↓, IL-10, IL-4Rɑ, SOCS3 ↑ in isolated microglia at 4 h;  mRNA: iNOS, IL-1β, Ym-1 ↑, IL-10, IL-4Rɑ, SOCS3 ↑ in isolated microglia at 24 h[59] |
|  | |  | | | LPS (0.5 mg/kg, *i.p.*), adult C57BL/6 CX3CR1^−/−^ mice (3–6 m) | | | | | | | OFT, TST | | Iba-1^+^↑ in PFC and HIP;  TRP↓, KYN ↑; TRP ↓ in plasma;  5-HIAA/5-HT, 3-HK/TRP ↑ in brain at 72 h[60] |
|  | |  | | | Mixed stress during adolescence (PND37–48) ＋ LPS (250 μg/kg, *i.p.* in adulthood), male and female Wistar rats | | | | | | | — | | mRNA: IL-1β, TNF-α , IκBα, CD11b, iNOS ↑ in HIP; CORT ↑ in plasma [61] |
|  | |  | | | LPS (1 μL, 5mg/mL, i.c.v.), male C57Bl/6J mice (12 weeks) | | | | | | | OFT, SPT | | Iba-1^+^↑ in HIP (hilus, CA1, CA3)[62] |
|  | |  | | | LPS (5 mg/kg, *i.p.*), Male C57BL/6 mice (8 weeks, 20–25 g) | | | | | | | Rotarod test, NORT, TST, FST, SPT | | Iba-1^+^↑;  ELISA: IL-1β, IL-18, TNF-α ↑, IL-10 ↓;  Proteins: NLRP3, ASC, caspase-1 ↑ in HIP;  ELISA: IL-1β, TNF-α ↑ in serum[63] |
|  | |  | | | LPS (100 ng/mL, *i.e.*) for 3 d, male SD rats (300–320g) | | | | | | | SPT, TST | | TNF-α^+^ , p-p38^+^↑ in habenula[64] |
|  | |  | | | LPS (100 ng/mL), primary cultures of microglial | | | | | | | — | | mRNA: CD40, MHC II, IL-1β, IL-18, IL-6, TNF-α, CCL2, NO, ROS ↑ , IL-10 , TGF-β, IGF-1 ↓;  Proteins: TLR4 ↑, ERK1/2, p65-NF-κB ↑ , p-INK/JNK ↑, IκB ↓, p-p38/p38 ↑, NLRP3, caspase-1, ASC ↑[65] |
|  | |  | | | LPS (1 mg/kg) for 5 d, adult male C57BL/6J mice (6–8 weeks, 25–30g) | | | | | | | SPT, TST, FST | | Iba-1^+^;  Proteins: iNOS, IL-6, IL-1β, MCP-1,TNF-α , HECTD1 ↓ in HIP  circDYM ↓ in plasma and brain[66] |
|  | |  | | | LPS (1 mg/kg, *i.p.*), CX3CR1CreER mice, Dlg1flox/flox mice | | | | | | | OFT,EMP,  TST, FST | | Iba-1^+^, iNOS^+^↑;  ELISA: TNF-α, IL-6;  mRNA: TNF-α, IL-6, IL-1β ↑ in HIP[67] |
|  | |  | | | LPS(0.83 mg/kg, *i.p.*), adult male Kunming mice (8–10 weeks, 30–50 g) | | | | | | | OFT, EMP, TST | | IL-1β, IL-10 ↑ in serum;  5-HT, NE ↓, 5-HIAA/5-HT, MHPG/NE ↑, DOPAC/DA ↓ in HIP[68] |

↑ upregulated; ↓ downregulated; (–) no significant difference.

**References**

1. Arauchi R, Hashioka S, Tsuchie K, Miyaoka T, Tsumori T, Limoa E, et al. Gunn rats with glial activation in the hippocampus show prolonged immobility time in the forced swimming test and tail suspension test. Brain Behav. 2018;8:e01028.

2. Puga DA, Tovar CA, Guan Z, Gensel JC, Lyman MS, McTigue DM, et al. Stress exacerbates neuron loss and microglia proliferation in a rat model of excitotoxic lower motor neuron injury. Brain Behav Immun. 2015;49:246–54.

3. MacDowell KS, Caso JR, Martín-Hernández D, Moreno BM, Madrigal JLM, Micó JA, et al. The Atypical Antipsychotic Paliperidone Regulates Endogenous Antioxidant/Anti-Inflammatory Pathways in Rat Models of Acute and Chronic Restraint Stress. Neurotherapeutics. 2016;13:833–43.

4. Walter TJ, Vetreno RP, Crews FT. Alcohol and Stress activation of microglia and neurons: brain regional effects. Alcohol Clin Exp Res. 2017;41:2066–81.

5. Sugama S, Takenouchi T, Hashimoto M, Ohata H, Takenaka Y, Kakinuma Y. Stress-induced microglial activation occurs through β-adrenergic receptor: noradrenaline as a key neurotransmitter in microglial activation. J Neuroinflammation. 2019;16:266.

6. Iwata M, Ishida H, Kaneko K, Shirayama Y. Learned helplessness activates hippocampal microglia in rats: A potential target for the antidepressant imipramine. Pharmacol Biochem Behav. 2016;150:138–46.

7. Wachholz S, Knorr A, Mengert L, Plümper J, Sommer R, Juckel G, et al. Interleukin-4 is a participant in the regulation of depressive-like behavior. Behavioural brain research. 2017;326:165–72.

8. Han L, Wang LY, Tang S, Yuan L, Wu SY, Du XZ, et al. ITGB4 deficiency in bronchial epithelial cells directs airway inflammation and bipolar disorder-related behavior. J Neuroinflammation. 2018;15:246.

9. Worthen RJ, Garzon Zighelboim SS, Torres Jaramillo CS, Beurel E. Anti-inflammatory IL-10 administration rescues depression-associated learning and memory deficits in mice. J Neuroinflammation. 2020;17:246.

10. Burke NN, Geoghegan E, Kerr DM, Moriarty O, Finn DP, Roche M. Altered neuropathic pain behaviour in a rat model of depression is associated with changes in inflammatory gene expression in the amygdala. Genes Brain Behav. 2013;12:70513.

11. Rinwa P, Kumar A. Quercetin suppress microglial neuroinflammatory response and induce antidepressent-like effect in olfactory bulbectomized rats. Neuroscience. 2013;255:86–98.

12. Burke NN, Kerr DM, Moriarty O, Finn DP, Roche M. Minocycline modulates neuropathic pain behaviour and cortical M1-M2 microglial gene expression in a rat model of depression. Brain Behav Immun. 2014;42:147–56.

13. Takahashi K, Nakagawasai O, Nemoto W, Kadota S, Isono J, Odaira T, et al. Memantine ameliorates depressive-like behaviors by regulating hippocampal cell proliferation and neuroprotection in olfactory bulbectomized mice. Neuropharmacology. 2018;137:141–55.

14. Yan L, Gu MQ, Yang ZY, Xia J, Li P, Vasar E, et al. Endogenous n-3 PUFAs attenuated olfactory bulbectomy-induced behavioral and metabolomic abnormalities in Fat-1 mice. Brain Behav Immun. 2021;96:143–53.

15. Ong LK, Zhao Z, Kluge M, TeBay C, Zalewska K, Dickson PW, et al. Reconsidering the role of glial cells in chronic stress-induced dopaminergic neurons loss within the substantia nigra? Friend or foe? Brain Behav Immun. 2017;60:117–25.

16. Tynan RJ, Naicker S, Hinwood M, Nalivaiko E, Buller KM, Pow DV, et al. Chronic stress alters the density and morphology of microglia in a subset of stress-responsive brain regions. Brain Behav Immun. 2010;24:1058–68.

17. Park JH, Yoo KY, Lee CH, Kim IH, Shin BN, Choi JH, et al. Comparison of glucocorticoid receptor and ionized calcium-binding adapter molecule 1 immunoreactivity in the adult and aged gerbil hippocampus following repeated restraint stress. Neurochem Res. 2011;36:1037–45.

18. Yoo KY, Lee CH, Park JH, Hwang IK, Park OK, Kwon S-H, et al. Antioxidant enzymes are differently changed in experimental ischemic hippocampal CA1 region following repeated restraint stress. J Neurol Sci. 2011;302:33–42.

19. Hinwood M, Morandini J, Day TA, Walker FR. Evidence that microglia mediate the neurobiological effects of chronic psychological stress on the medial prefrontal cortex. Cereb Cortex. 2012;22:1442–54.

20. Kopp BL, Wick D, Herman JP. Differential effects of homotypic vs. heterotypic chronic stress regimens on microglial activation in the prefrontal cortex. Physiol Behav. 2013;122:246–52.

21. Gerecke KM, Kolobova A, Allen S, Fawer JL. Exercise protects against chronic restraint stress-induced oxidative stress in the cortex and hippocampus. Brain Res. 2013;1509:66–78.

22. Couch Y, Anthony DC, Dolgov O, Revischin A, Festoff B, Santos AI, et al. Microglial activation, increased TNF and SERT expression in the prefrontal cortex define stress-altered behaviour in mice susceptible to anhedonia. Brain Behav Immun. 2013;29:136–46.

23. Orellana JA, Moraga-Amaro R, Díaz-Galarce R, Rojas S, Maturana CJ, Stehberg J, et al. Restraint stress increases hemichannel activity in hippocampal glial cells and neurons. Front Cell Neurosci. 2015;9:102.

24. Guo Y, Xie JP, Li X, Yuan Y, Zhang LC, Hu WY, et al. Antidepressant effects of *Rosemary*extracts associate with anti-inflammatory effect and rebalance of gut microbiota. Front Pharmacol. 2018;9:1126.

25. Feng X, Zhao Y, Yang T, Song M, Wang C, Yao Y, et al. Glucocorticoid-driven NLRP3 inflammasome activation in hippocampal microglia mediates chronic stress-induced depressive-like behaviors. Front Mol Neurosci. 2019;12:210.

26. Mao ZF, Ouyang SH, Zhang QY, Wu YP, Wang GE, Tu LF, et al. New insights into the effects of caffeine on adult hippocampal neurogenesis in stressed mice: Inhibition of CORT-induced microglia activation. FASEB J. 2020;34:10998–1014.

27. Wu D, Zhang GC, Zhao CY, Yang Y, Miao ZG, Xu XS. Interleukin-18 from neurons and microglia mediates depressive behaviors in mice with post-stroke depression. Brain Behav Immun. 2020;88:411–20.

28. Kodali M, Hattiangady B, Shetty GA, Bates A, Shuai B, Shetty AK. Curcumin treatment leads to better cognitive and mood function in a model of Gulf War Illness with enhanced neurogenesis, and alleviation of inflammation and mitochondrial dysfunction in the hippocampus. Brain Behav Immun. 2018;69:499–514.

29. Zhou SH, Chen SS, Xie WX, Guo XX, Zhao JF. Microglia polarization of hippocampus is involved in the mechanism of Apelin-13 ameliorating chronic water immersion restraint stress-induced depression-like behavior in rats. Neuropeptides. 2020;81:102006.

30. Piirainen S, Chithanathan K, Bisht K, Piirsalu M, Savage JC, Tremblay ME, et al. Microglia contribute to social behavioral adaptation to chronic stress. Glia. 2021;69:2459–73.

31. Guo Y, Xie JP, Zhang LC, Yang LL, Ma JQ, Bai YF, et al. Ginsenoside Rb1 exerts antidepressant-like effects via suppression inflammation and activation of AKT pathway. Neurosci Lett. 2021;744:135561.

32. Zhou YF, Yan MZ, Pan RL, Wang Z, Tao X, Li CC, et al. *Radix Polygalae* extract exerts antidepressant effects in behavioral despair mice and chronic restraint stress-induced rats probably by promoting autophagy and inhibiting neuroinflammation. J Ethnopharmacol. 2021;265:113317.

33. Brachman RA, Lehmann ML, Maric D, Herkenham M. Lymphocytes from chronically stressed mice confer antidepressant-like effects to naive mice. J Neurosci. 2015;35:1530–8.

34. Lehmann ML, Cooper HA, Maric D, Herkenham M. Social defeat induces depressive-like states and microglial activation without involvement of peripheral macrophages. J Neuroinflammation. 2016;13:224.

35. Ito N, Hirose E, Ishida T, Hori A, Nagai T, Kobayashi Y, et al. Kososan, a Kampo medicine, prevents a social avoidance behavior and attenuates neuroinflammation in socially defeated mice. J Neuroinflammation. 2017;14:98.

36. Tong LJ, Gong Y, Wang P, Hu WF, Wang JL, Chen Z, et al. Microglia loss contributes to the development of major depression induced by different types of chronic stresses. Neurochem Res. 2017;42:2698–711.

37. Lehmann ML, Weigel TK, Cooper HA, Elkahloun AG, Kigar SL, Herkenham M. Decoding microglia responses to psychosocial stress reveals blood-brain barrier breakdown that may drive stress susceptibility. Sci Rep. 2018;8:11240.

38. McAllister BB, Wright DK, Wortman RC, Shultz SR, Dyck RH. Elimination of vesicular zinc alters the behavioural and neuroanatomical effects of social defeat stress in mice. Neurobiol Stress. 2018;9:199–213.

39. Tian T, Xu B, Qin YH, Fan L, Chen JJ, Zheng P, et al. Clostridium butyricum miyairi 588 has preventive effects on chronic social defeat stress-induced depressive-like behaviour and modulates microglial activation in mice. Biochem Biophys Res Commun. 2019;516(2):430–6.

40. Ferle V, Repouskou A, Aspiotis G, Raftogianni A, Chrousos G, Stylianopoulou F, et al. Synergistic effects of early life mild adversity and chronic social defeat on rat brain microglia and cytokines. Physiol Behav. 2020;215:112791.

41. Nozaki K, Ito H, Ohgidani M, Yamawaki Y, Sahin EH, Kitajima T, et al. Antidepressant effect of the translocator protein antagonist ONO-2952 on mouse behaviors under chronic social defeat stress. Neuropharmacology. 2020;162:107835.

42. Espinosa-Garcia C, Atif F, Yousuf S, Sayeed I, Neigh GN, Stein DG. Progesterone attenuates stress-induced NLRP3 inflammasome activation and enhances autophagy following ischemic brain injury. Int J Mol Sci. 2020;21:3740.

43. Zhang K, Yang C, Chang LX, Sakamoto A, Suzuki T, Fujita Y, et al. Essential role of microglial transforming growth factor-β1 in antidepressant actions of (*R*)-ketamine and the novel antidepressant TGF-β1. Transl Psychiatry. 2020;10:32.

44. Pearson-Leary J, Zhao C, Bittinger K, Eacret D, Luz S, Vigderman AS, et al. The gut microbiome regulates the increases in depressive-type behaviors and in inflammatory processes in the ventral hippocampus of stress vulnerable rats. Mol Psychiatry. 2020;25:1068–79.

45. Beins EC, Beiert T, Jenniches I, Hansen JN, Leidmaa E, Schrickel JW, et al. Cannabinoid receptor 1 signalling modulates stress susceptibility and microglial responses to chronic social defeat stress. Transl Psychiatry. 2021;11:164.

46. Peng ZL, Zhang C, Yan L, Zhang YP, Yang ZY, Wang JJ, et al. EPA is more effective than DHA to improve depression-like behavior, glia cell dysfunction and hippcampal apoptosis signaling in a chronic stress-induced rat model of depression. Int J Mol Sci. 2020;21:1769.

47. Zhao QY, Wu XH, Yan S, Xie XF, Fan YH, Zhang JQ, et al. The antidepressant-like effects of pioglitazone in a chronic mild stress mouse model are associated with PPARγ-mediated alteration of microglial activation phenotypes. J Neuroinflammation. 2016;13:259.

48. Guo LT, Wang SQ, Su J, Xu LX, Ji ZY, Zhang RY, et al. Baicalin ameliorates neuroinflammation-induced depressive-like behavior through inhibition of toll-like receptor 4 expression via the PI3K/AKT/FoxO1 pathway. J Neuroinflammation. 2019;16(1):95.

49. Zhang LJ, Tang MM, Xie XF, Zhao QY, Hu N, He H, et al. Ginsenoside Rb1 induces a pro-neurogenic microglial phenotype via PPARγ activation in male mice exposed to chronic mild stress. J Neuroinflammation. 2021;18(1):171.

50. Zhang JQ, Wu XH, Feng Y, Xie XF, Fan YH, Yan S, et al. Salvianolic acid B ameliorates depressive-like behaviors in chronic mild stress-treated mice: involvement of the neuroinflammatory pathway. Acta Pharmacol Sin. 2016;37(9):1141–53.

51. Wang YL, Wu HR, Zhang SS, Xiao HL, Yu J, Ma YY, et al. Catalpol ameliorates depressive-like behaviors in CUMS mice via oxidative stress-mediated NLRP3 inflammasome and neuroinflammation. Transl Psychiatry. 2021;11:353.

52. Su WJ, Zhang T, Jiang C-L, Wang W. Clemastine alleviates depressive-like behavior through reversing the imbalance of microglia-related pro-inflammatory state in mouse hippocampus. Front Cell Neurosci. 2018;12:412.

53. Lee JS, Kim WY, Jeon YJ, Lee SB, Lee DS, Son CG. Antidepressant-like activity of *Myelophil* attenuation of microglial-mediated neuroinflammation in mice undergoing unpredictable chronic mild stress. Front Pharmacol. 2019;10:683.

54. Wang R, Wang W, Xu JJ, Liu DX, Jiang H, Pan F. Dynamic effects of early adolescent stress on depressive-like behaviors and expression of cytokines and JMJD3 in the prefrontal cortex and hippocampus of rats. Front Psychiatry. 2018;9:471.

55. Zhu HX, Cheng LJ, Ou Yang RW, Li YY, Liu J, Dai D, et al. Reduced amygdala microglial expression of brain-derived neurotrophic factor and tyrosine kinase receptor B (TrkB) in a rat model of poststroke depression. Med Sci Monit. 2020;26:e926323.

56. Liu CP, Zhong M, Sun JX, He J, Gao Y, Qin FX. miR‑146a reduces depressive behavior byinhibiting microglial activation. Mol Med Rep. 2021;23:463.

57. Zhang L, Zhang L, Sui RB. Ganoderic acid A-mediated modulation of microglial polarization is involved in depressive-like behaviors and neuroinflammation in a rat model of post-stroke depression. Neuropsychiatr Dis Treat. 2021;17:2671–81.

58. Corona AW, Huang Y, O'Connor JC, Dantzer R, Kelley KW, Popovich PG, et al. Fractalkine receptor (CX3CR1) deficiency sensitizes mice to the behavioral changes induced by lipopolysaccharide. J Neuroinflammation. 2010;7:93.

59. Fenn AM, Henry CJ, Huang Y, Dugan A, Godbout JP. Lipopolysaccharide-induced interleukin (IL)-4 receptor-α expression and corresponding sensitivity to the M2 promoting effects of IL-4 are impaired in microglia of aged mice. Brain Behav Immun. 2012;26(5):766–77.

60. Corona AW, Norden DM, Skendelas JP, Huang Y, O'Connor JC, Lawson M, et al. Indoleamine 2,3-dioxygenase inhibition attenuates lipopolysaccharide induced persistent microglial activation and depressive-like complications in fractalkine receptor (CX(3)CR1)-deficient mice. Brain Behav Immun. 2013;31:134–42.

61. Pyter LM, Kelly SD, Harrell CS, Neigh GN. Sex differences in the effects of adolescent stress on adult brain inflammatory markers in rats. Brain Behav Immun. 2013;30:88–94.

62. van Buel EM, Bosker FJ, van Drunen J, Strijker J, Douwenga W, Klein HC, et al. Electroconvulsive seizures (ECS) do not prevent LPS-induced behavioral alterations and microglial activation. J Neuroinflammation. 2015;12:232.

63. Zhu W, Cao FS, Feng J, Chen HW, Wan JR, Lu Q, et al. NLRP3 inflammasome activation contributes to long-term behavioral alterations in mice injected with lipopolysaccharide. Neuroscience. 2017;343:77–84.

64. Zhao YW, Pan YQ, Tang MM, Lin WJ. Blocking p38 signaling reduces the activation of pro-inflammatory cytokines and the phosphorylation of p38 in the habenula and reverses depressive-like behaviors induced by neuroinflammation. Front Pharmacol. 2018;9:511.

65. Ślusarczyk J, Trojan E, Głombik K, Piotrowska A, Budziszewska B, Kubera M, et al. Targeting the NLRP3 inflammasome-related pathways via tianeptine treatment-suppressed microglia polarization to the M1 phenotype in lipopolysaccharide-stimulated cultures. Int J Mol Sci. 2018;19:1965.

66. Zhang Y, Du LF, Bai Y, Han B, He CC, Gong L, et al. CircDYM ameliorates depressive-like behavior by targeting miR-9 to regulate microglial activation via HSP90 ubiquitination. Mol Psychiatry. 2020;25(6):1175–90.

67. Peng ZX, Li XH, Li J, Dong Y, Gao YH, Liao YJ, et al. Dlg1 knockout inhibits microglial activation and alleviates lipopolysaccharide-induced depression-like behavior in mice. Neurosci Bull. 2021;37: 1671–82.

68. Li P, Zhang FC, Li YJ, Zhang C, Yang ZY, Zhang YP, et al. Isoginkgetin treatment attenuated lipopolysaccharide-induced monoamine neurotransmitter deficiency and depression-like behaviors through downregulating p38/NF-κB signaling pathway and suppressing microglia-induced apoptosis. J Psychopharmacol. 2021;35:1285–99.
